# Transcriptomic Identification of Drought-Related Genes and SSR Markers in Sudan Grass Based on RNA-Seq

**DOI:** 10.3389/fpls.2017.00687

**Published:** 2017-05-04

**Authors:** Yongqun Zhu, Xia Wang, Linkai Huang, Chaowen Lin, Xinquan Zhang, Wenzhi Xu, Jianhua Peng, Zhou Li, Haidong Yan, Fuxiang Luo, Xie Wang, Li Yao, Dandan Peng

**Affiliations:** ^1^Department of Grassland Science, Animal Science and Technology College, Sichuan Agricultural UniversityChengdu, China; ^2^Soil and Fertilizer Research Institute, Sichuan Academy of Agricultural SciencesChengdu, China; ^3^Sichuan Academy of Agricultural SciencesChengdu, China

**Keywords:** Sudan grass, next-generation sequencing, differentially expressed genes, simple sequence repeat markers, PEG

## Abstract

Sudan grass (*Sorghum sudanense*) is an annual warm-season gramineous forage grass that is widely used as pasture, hay, and silage. However, drought stress severely impacts its yield, and there is limited information about the mechanisms of drought tolerance in Sudan grass. In this study, we used next-generation sequencing to identify differentially expressed genes (DEGs) in the Sudan grass variety Wulate No.1, and we developed simple sequence repeat (SSR) markers associated with drought stress. From 852,543,826 raw reads, nearly 816,854,366 clean reads were identified and used for analysis. A total of 80,686 unigenes were obtained via *de novo* assembly of the clean reads including 45,065 unigenes (55.9%) that were identified as coding sequences (CDSs). According to Gene Ontology analysis, 31,444 unigenes were annotated, 11,778 unigenes were identified to 25 categories in the clusters of orthologous groups of proteins (KOG) classification, and 11,223 unigenes were assigned to 280 Kyoto Encyclopedia of Genes and Genomes (KEGG) pathways. Additionally, there were 2,329 DEGs under a short-term of 25% polyethylene glycol (PEG) treatment, while 5,101 DEGs were identified under the long-term of 25% PEG treatment. DEGs were enriched in pathways of carbon fixation in photosynthetic organisms and plant hormone signal transduction which played a leading role in short-term of drought stress. However, DEGs were mainly enriched in pathway of plant hormone signal transduction that played an important role under long-term of drought stress. To increase accuracy, we excluded all the DEGs of all controls, specifically, five DEGs that were associated with high PEG concentrations were found through RNA-Seq. All five genes were up-regulated under drought stress, but the functions of the genes remain unclear. In addition, we identified 17,548 SSRs obtained from 80,686 unigenes. The newly identified drought tolerance DEGs will contribute to transgenic breeding efforts, while SSRs developed from high-throughput transcriptome data will facilitate marker-assisted selection for all traits in Sudan grass.

## Introduction

Global climate change has increased the incidence of drought worldwide across successive years (Shanker et al., 2014), and it has had many negative effects on plant growth such as leaf rolling, growth inhibition, and death (Mutisya et al., 2010), thus severely affecting agriculture. Sudan grass (*Sorghum sudanense*) is an annual warm-season gramineous forage grass (Smith et al., 1982; Zhan et al., 2008). Because of its favorable yield, superior value, fast regrowth, superior disease, and pest resistance, and tolerance to abiotic stress, Sudan grass is widely grown in pastures and used as hay and silage throughout the world (Rangaswami Ayyangar and Ponnaiya, 1939; Summer et al., 1965; Zamfir et al., 2001; Al-Suhaibani, 2006). It is distributed throughout China as a typical grass for livestock, aquaculture foods, and protecting fishing ponds (Wei et al., 2008; Chen et al., 2009), especially in arid and semiarid regions (Awad et al., 2013). However, exposure to drought stress for long time periods can affect forage yield and quality. Bibi et al. (2010) showed that drought stress could decrease Sudan grass yields. Therefore, it is important to study drought tolerance mechanisms in Sudan grass.

Drought stress causes osmotic and oxidative stress, which decreases membrane stability, leading to cell death (Wang et al., 2003). In order to cope with drought stress, plants have developed comprehensive mechanisms such as metabolic alteration, signal transduction, and differential gene expression (Shanker et al., 2014). Although various breeding approaches have been used to alleviate damage caused by drought stress in plants, genetic engineering has been more effective than other approaches. However, genetic engineering requires identifying genes with expression patterns regulated by drought stress.

The next-generation sequencing (NGS) technique known as RNA sequencing (RNA-Seq) is a cost-efficient tool for sequencing the full transcriptomes of both model and non-model species. RNA-Seq has been successfully used in many plants such as *Brachypodium sylvaticum* (Fox et al., 2013), *Sorghum sudanense* (Li et al., 2016), sugarcane (Cardoso-Silva et al., 2014), pepper (Ashrafi et al., 2012), orchardgrass (Huang et al., 2015), *Hemarthria* (Huang X. et al., 2016), and annual ryegrass (Pan et al., 2016). Transcriptome data has been used in biological studies worldwide in order to better understand biological processes (Surget-Groba and Montoya-Burgos, 2010), and it has especially been applied to studying the responses of gene expression to various stresses (Kreps et al., 2002). Shinozaki and Yamaguchi-Shinozaki (2007) reported that plants transformed with drought-inducible genes exhibited improved stress tolerance. Similarly, Ashraf (2010) noted that some genes are overexpressed, thereby inducing damage caused by drought stress, which are thus well utilized to improve the tolerance of plants to drought stress. However, effectively no published reports have used RNA-Seq to analyze the regulation of gene expression by the drought stress in Sudan grass.

Marker-assisted selection (MAS) breeding is as important as genetic engineering (Ashraf, 2010). SSRs (simple sequence repeats), AFLPs (amplified fragment length polymorphisms), RAPDs (randomly amplified polymorphic DNAs), and RFLPs (restriction fragment length polymorphisms) have been used as efficient markers to analyze genetic diversity (Billot et al., 2013). Because SSRs are highly polymorphic and adaptable across species, many researchers have used them to examine genetic diversity (Smith et al., 2000; Menz et al., 2002; Geleta et al., 2006), construct genetic maps (Wu and Huang, 2006), investigate the genetic relationships among populations (Ali et al., 2008), and identify quantitative trait loci (QTLs) for important agronomic traits (Sanchez et al., 2002; Mace and Jordan, 2011; Wang et al., 2012; Upadhyaya et al., 2012). However, few SSR markers have been developed for use in Sudan grass.

In this study, we used RNA-Seq, a powerful NGS-based technique, to study transcription profiles of Sudan grass. The main goals of this study were (1) to develop SSR markers associated with drought-tolerance genes in the Sudan grass variety Wulate No. 1 and (2) to identify differently expressed genes (DEGs) under drought stress. This study provides more information about the molecular mechanisms of drought stress in Sudan grass, thereby contributing to future transgenic breeding efforts in addition to providing markers for MAS.

## Materials and methods

### Plant material and RNA isolation

Seeds of the Sudan grass variety Wulate No.1 (Barenbrug Co., Chengdu, China) were sown in sand-culture pots that were placed in controlled growth champers set to 25°C for 12-h days and 22°C for 12-h nights. After seeds had germinated in water, 1/2 strength Hoagland's solution was used to cultivate seedlings. After 20 days, seedlings were subjected to polyethylene glycol (PEG) stress as a means of inducing drought stress. The plants were divided into two treatments: (1) plants in three pots (three replicates) were subjected to 25% PEG dissolved in 1/2 strength Hoagland's solution (drought stress); (2) the other three pots were subjected to just 1/2 strength Hoagland's solution (control). The leaves were harvested at 0, 3, and 6 days and stored in a −80°C freezer prior to RNA extraction. L_0_1, L_3_1, and L_6_1 were non-stressed controls that were collected at 0, 3, and 6 day, respectively. L_3_2 and L_6_2 were drought-stressed treatments that were collected at 3 and 6 day, respectively. Therefore, 5 treatments were set and each treatment had 3 replicates. A total of 15 samples were collected for RNA sequencing.

**Figure 1:**
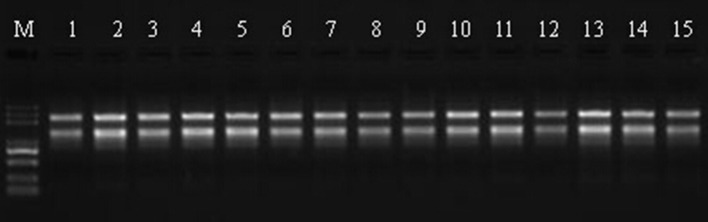
**RNA validation of Sudan grass samples by 1% agarose gel electrophoresis**. M represents the marker, lanes 1–3 correspond to the three replicates of Wulate No.1 at 0 day in 0% PEG, lanes 4–6 correspond to the three replicates of Wulate No.1 at 3 day in 0% PEG, lanes 7–9 correspond to the three replicates of Wulate No.1 at 3 day in 25% PEG, lanes 10–12 correspond to the three replicates of Wulate No.1 at 6 day in 0% PEG, and lanes 13–15 correspond to the three replicates of Wulate No.1 at 6 day in 25% PEG.

**Figure 2:**
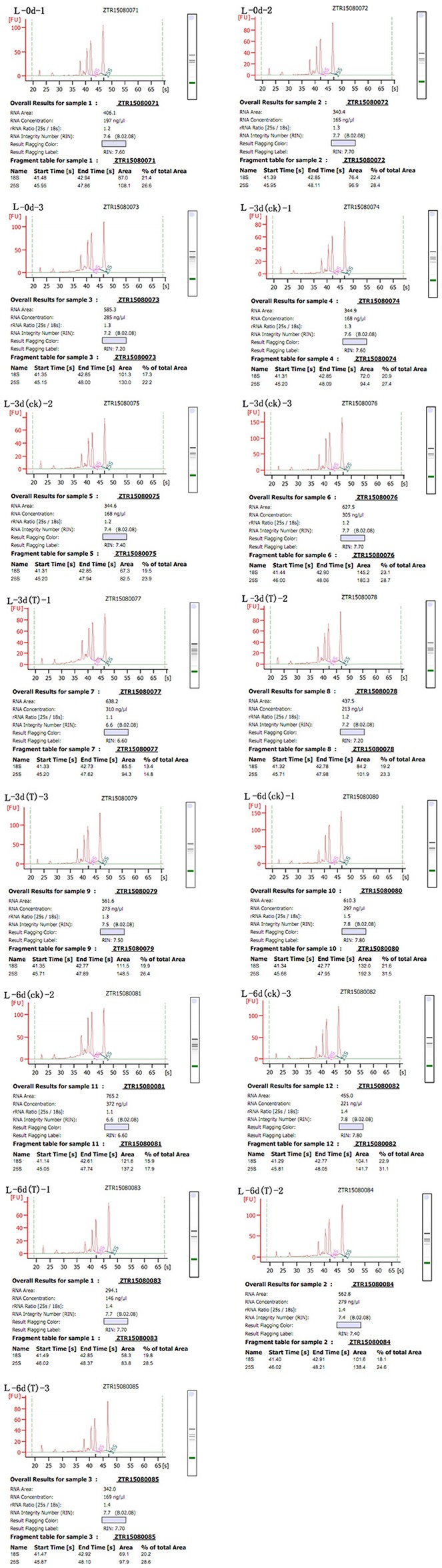
**Sudan grass RNA sample quality**. The labeling scheme for each of the Wulate No.1 RNA samples is as follows: L-0d-1 through L-0d-3, the three replicates at day 0 in 0% PEG; L-3d(ck)-1 through L-3d(ck)-3, the three replicates at day 3 in 0% PEG; L-3d(T)-1 through L-3d(T)-3, the three replicates at day 3 in 25% PEG; L-6d(ck)-1 through L-6d(ck)-3, the three replicates at day 6 in 0% PEG; and L-6d(T)-1 through L-6d(T)-3, the three replicates at day 6 in 25% PEG.

Total RNA was extract from leaves using the RNeasy Plant Mini Kit (Qiagen, Valencia, CA, USA) according to the manufacturer's instructions and then loaded onto and electrophoresed through a 1% agarose gel to check the degradation and contamination of the RNA (Figure 1). The quantity, concentration, and quality of the total RNA were examined using an RNA Nano 6000 kit for the Agilent 2100 Bioanalyzer 2100 System (Agilent Technologies, CA, USA). The integrity of the RNAs is shown in Figure 2.

### Preparation and sequencing of the cDNA library

We used 3 μg of RNA per sample as genetic material for preparation, and mRNAs were purified from total RNA using poly-T oligo-attached magnetic beads. These fragments were used as templates in a reaction carried out by divalent cations in a NEBNext First Strand Synthesis Reaction Buffer (5×; New England BioLabs, Ipswitch, MA, USA). The first cDNA strand was synthesized using random hexamer primers and transcribed with RNase H^−^. The second strand cDNA was synthesized using DNA polymerase I and RNase H. NEBNext Adaptor (New England BioLabs) were prepared for hybridization after blunt 3′ ends were cut via exonuclease/polymerase activities. Then, we purified cDNA with the AMPure XP system (Beckman Coulter, Brea, CA USA) to select 150–200 bp fragments. The products were generated using 3 μL of USER Enzyme (New England BioLabs) size-selected for adaptor-ligated cDNA at 37°C for 15 min followed by 5 min at 95°C and amplified via PCR using Universal PCR primers and index(X) primers. The PCR was performed using Phusion High-Fidelity DNA polymerase (New England BioLabs). Before PCR product sequencing, samples were index-coded for clustering under the TruSeq PE Cluster Kit v3-cBot-HS (Illumia, San Diego, CA, USA). Finally, the amplified products were purified using the AMPure XP system, and library quality was assessed on the Agilent Bioanalyzer 2100 system (Agilent Technologies, Santa Clara, CA, USA) to create the final cDNA libraries. Sequencing libraries were generated using NEBNext® Ultra™ RNA Library Prep Kit for Illumina® (New England BioLabs) following the manufacturer's recommendations. In total, there were 15 cDNA libraries were constructed of Sudan grass.

### Data analysis

The sequencing-derived image data were transformed into raw reads. To ensure high quality transcriptomic data, the raw reads were cleaned using in-house Perl scripts that remove adapter sequences, poly-N repeats, and low quality reads, whilst calculating the Q20, Q30, GC-content, and sequence duplication level of the clean data. The cleaned reads were then assembled by Trinity (Grabherr et al., 2011) with min_kmer_cov set to 2 as a default, and all other parameters used default settings.

The assembled sequences were BLASTed against NR (NCBI non-redundant protein sequences, NCBI blast 2.2.28+, e-value = 1e-5), NT (NCBI non-redundant nucleotide sequences, NCBI blast 2.2.28+, e-value = 1e-5), PFAM (Protein family, HMMER 3.0 package, hmmscan e-value = 0.01), KOG/COG (Clusters of Orthologous Groups of proteins, NCBI blast 2.2.28+, e-value = 1e-3), Swiss-Prot (a manually annotated and reviewed protein sequence database, NCBI blast 2.2.28+, e-value = 1e-5), KO (KEGG Ortholog database, KAAS [KEGG Automatic Annotation Server], e-value = 1e-10), and GO (Gene Ontology, Blast 2 GO v2.5, e-value = 1e-6; Götz et al., 2008) databases to determine gene functions of differentially expressed transcripts. Then, we predicted the coding sequences (CDSs) of the assembled unigenes in NR and Swiss-Prot. When similar unigenes were identified, the CDSs were translated into amino acid sequences later and open reading frames (ORFs) were extracted from the results; alternatively, ESTScan (3.0.3) was used to predict ORFs.

### Analysis and mapping of DEG reads and differential gene expression

Before mapping clean reads onto the reference genome with RSEM (Li and Dewey, 2011), the assembled Trinity transcript data was regarded as the reference sequence. The bowtie 2 mismatch parameter in RSEM was set to 0. The quantity of read counts for all genes was obtained from the mapping results and then normalized by FPKM (expected number of fragments per kilobase of transcript sequence per millions base pairs sequenced; Trapnell et al., 2010). Differential expression analysis of each sample was performed using the DESeq R package (1.10.1). DESeq provides statistical routines for detecting genes exhibiting differential expression with a negative binominal distribution model (Anders and Huber, 2010). To ensure the accuracy of the *P*-values, Benjamini and Hochberg's approach was used to control the false discovery rate (FDR), after which a *P* < 0.05 threshold was used to classify genes as differentially expressed. All DEGs were subjected to GO enrichment analysis. To adjust for gene length, a Wallenius non-central hyper-geometric distribution (Young et al., 2010) was applied in the GO enrichment analysis using the GOseq R package (Young et al., 2010). Next, KOBAS 2.0 (Mao et al., 2005) was utilized to test the statistical enrichment of DEGs in KEGG pathways, a database resource for understanding high-level functions within biological systems (Kanehisa et al., 2008). In KOBAS 2.0, a FDR ≤ 0.05 threshold was used for remarkable enrichment pathways, and the FDR parameter was set as BH in KOBAS 2.0. Finally, blastx was used to search the genome of a related species in the string database with the DEG sequences in order to obtain the protein–protein interactions. The results were then visualized in Cytoscape (Shannon et al., 2003).

### SSR detection and primer design

In order to identify and locate SSRs (Simple Sequence Repeats) from the unigenes, MISA 1.0 was used. It detected SSRs according to the following unit size and minimum repeat parameters: 1-10, 2-6, 3-5, 4-5, 5-5, and 6-5. SSR primers were designed using PRIMER 3 (2.3.4) with the following parameters: length range, 18–23, with 21 as optimal; PCR product size range, 100–300 bp; optimum annealing temperature, 55°C; and GC content, 40–60%, with 50% as optimal.

### Survey of SSR polymorphism

A total of 20 Sudan grass and sorghum accessions were used to identify SSR markers. We randomly chose 30 SSR primers to evaluate their polymorphism. DNA extractions were performed using the DNeasy Plant Mini Kit (Qiagen) from leaves of the 20 Sudan grass and sorghum accessions. PCR amplifications were conducted in a total reaction volume of 15 μL containing 1.5 μL of DNA, 0.5 μL of Golden DNA Polymerase (TIANGEN Biotech, Beijing, China), 7.5 μL of 2× Reaction Mix (TIANGEN), 0.6 μL of each primer, and 4.4 μL of ddH_2_O. The PCR reaction cycling profile was 94°C for 4 min followed by 35 cycles at 94°C for 40 s, 55–60°C for 40 s, 72°C for 1 min, and a final extension at 72°C for 10 min. PCR products were separated via gel electrophoresis on a 6% denaturing polyacrylamide gels at 350 V for 1 h alongside a 50-bp DNA marker used to assess product lengths. Then, we used 0.1% AgNO_3_ to stain the gel. Well amplified loci were identified using Gel Doc™ XR (Bio-Rad Laboratories Inc., Hercules, CA, USA).

## Results and discussion

### Sequence analysis and transcript assembly

**Figure 3:**
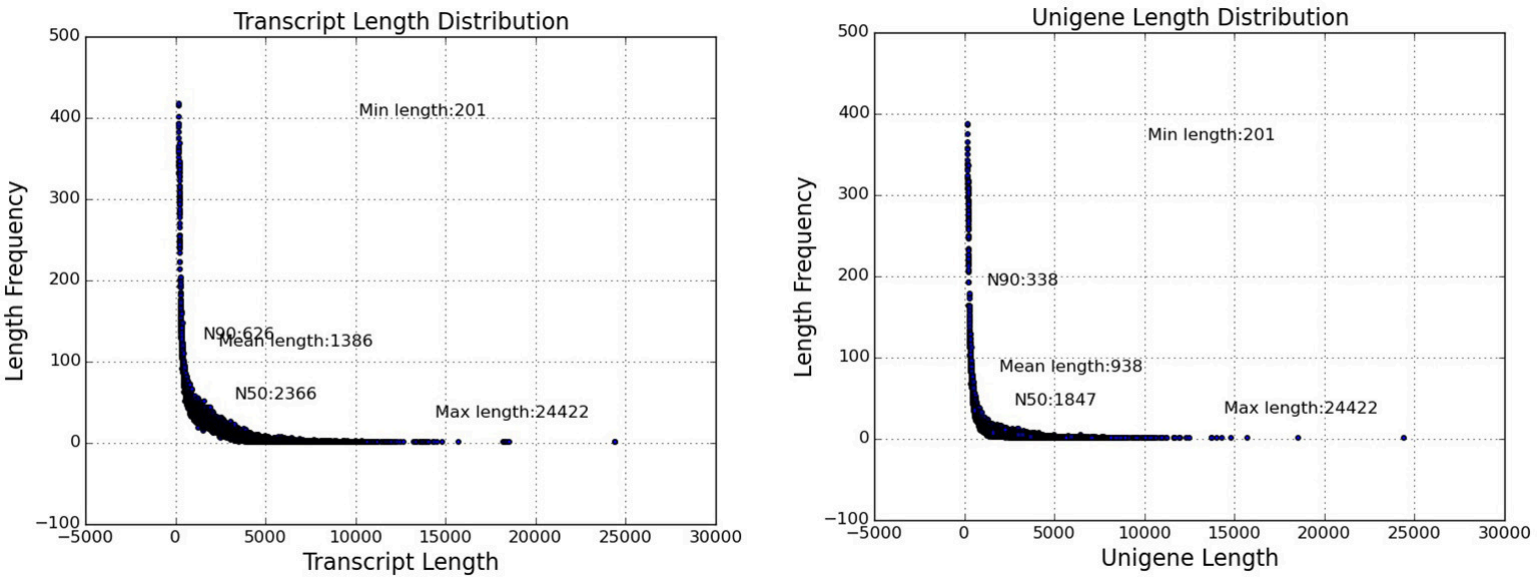
**Transcriptome and unigene length distribution of Sudan grass**.

Replicate cDNA libraries from Sudan grass leaf samples of plants grown under drought and control treatments (Supplementary Image 1) were constructed and sequenced on the Illumina HiSeq 2000 platform. A total of 852,543,826 raw reads were generated, and they have been deposited in the NCBI (National Center for Biotechnology Information) Short Read Archive (SRA: SRP095822). A total of 816,854,366 clean reads were identified after trimming adapters and filtering out low quality reads (Table 1). Using Trinity to further assemble the sequences, we obtained 149,395 contigs with a mean length of 1,386 bp, N50 of 2,366, and lengths of 201–24,422 bp, amounting to a total of 207,118,332 bp (Figure 3). The contigs were further assembled into 80,686 unigenes with a mean length of 938 bp and N50 of 1847 across a total of 75,697,479 bp (Figure 3). There are few Sudan grass ESTs in NCBI databases; however, Li et al. (2010) and Lu et al. (2009) used ESTs from *Sorghum bicolor* to explore SSRs in Sudan grass. Thus, the present results will serve as valuable genomic resources that will help identify more valuable SSRs.

**Table 1:** **Summary statistics of the Sudan grass transcriptome assemblies**.

**Sample**	**Raw reads**	**Clean reads**
L_0_1	168,623,898	165,220,518
L_3_1	173,980,764	169,869,984
L_3_2	171,496,394	163,559,358
L_6_1	183,220,324	173,103,162
L_6_2	155,222,446	145,101,344
Total	852,543,826	816,854,366

*L_0_1, L_3_1, and L_6_1 were non-stressed controls that were collected at 0, 3, and 6 day, respectively. L_3_2 and L_6_2 were drought-stressed treatments that were collected at 3 and 6 day, respectively (Each treatment had three replicates). The same below*.

### Gene function annotation

A total of 80,686 unigenes were annotated successfully. Totals of 38,919 unigenes (48.23%) showed significant similarity to the NR database, 46,064 (57.09%) to the NT database, 11,223 (13.9%) to the KO database, 24,436 (30.28%) to the Swiss-Prot database, 26,786 (33.19%) to the PFAM database, 31,444 (38.97%) to the GO database, and 11,778 (14.58%) to the KOG database. When the sequences were compared to all databases, only 5,892 unigenes (7.3%) were annotated in all, while all 52,315 unigenes (64.83%) were annotated in at least one database according to Blastx search (Table 2).

**Table 2:** **Unigene information annotated in different databases**.

**Database**	**Number of unigenes**	**Percentage (%)**
Annotated in NR	38,919	48.23
Annotated in NT	46,064	57.09
Annotated in KO	11,223	13.9
Annotated in Swiss-Prot	24,436	30.28
Annotated in PFAM	26,786	33.19
Annotated in GO	31,444	38.97
Annotated in KOG	11,778	14.59
Annotated in all Databases	5,892	7.3
Annotated in at least one Database	52,315	64.83
Total Unigenes	80,686	100

**Figure 4:**
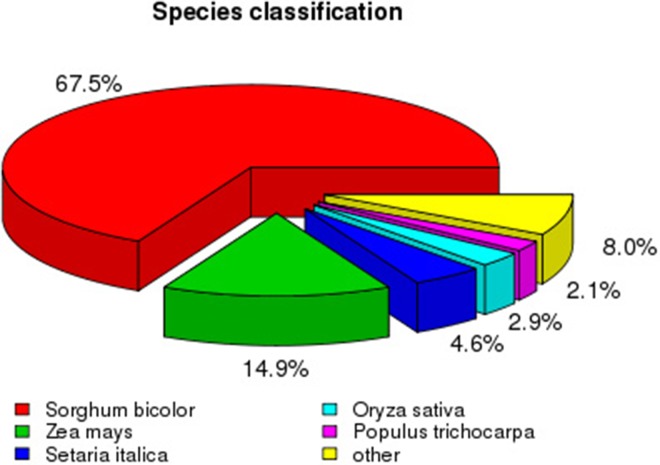
**Species distribution of all the unigene sequences**.

To analyze the conservation of sequences, we compared Sudan grass sequences to those from other species. The top match was *Sorghum bicolor* (65.7% sequence identity), followed by *Zea mays* (14.9%), *Setaria italica* (4.6%), and *Oryza sativa* (2.9%; Figure 4). As expected, more than 88% of sequences among all unigenes had a top match with sequences from plants in the family Poaceae.

**Figure 5:**
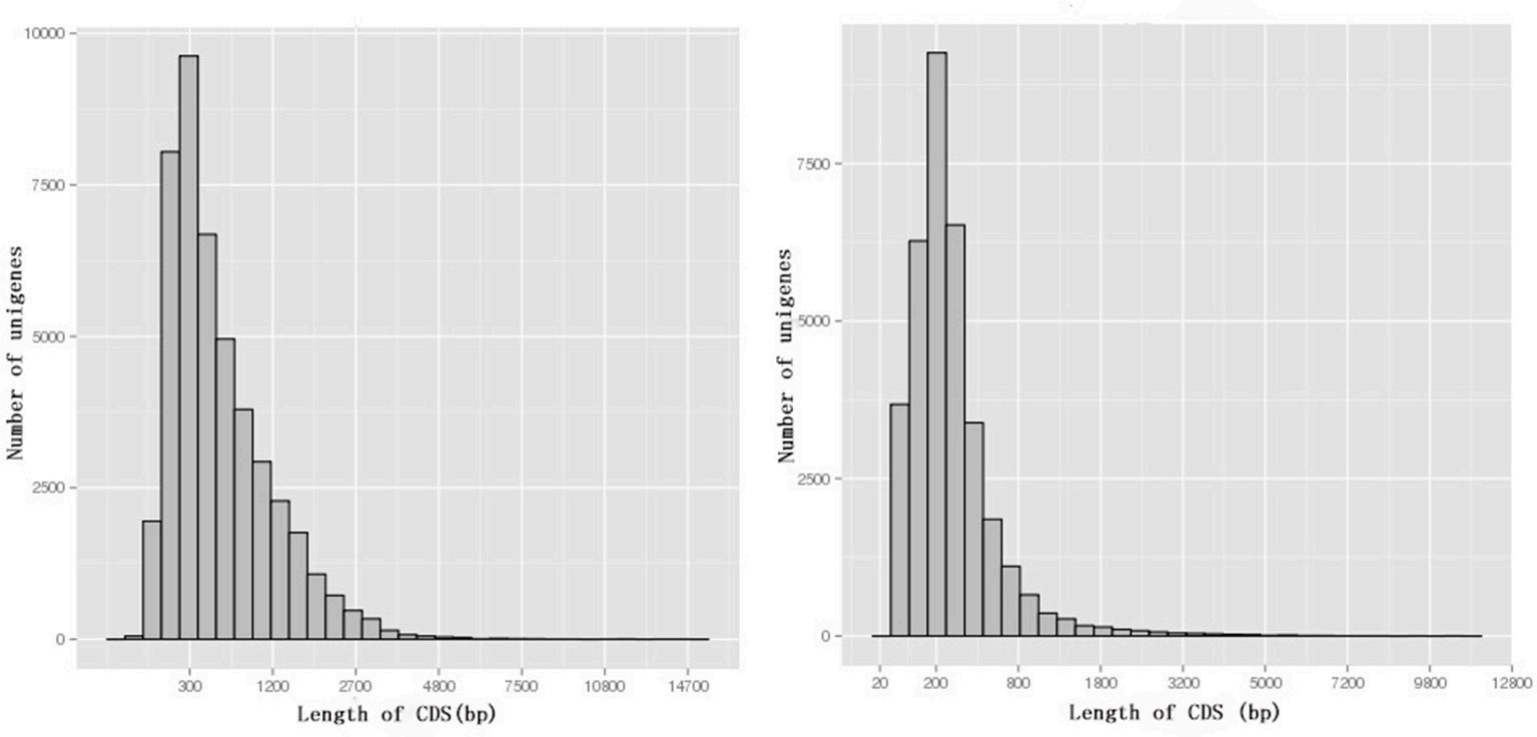
**The length distribution of CDSs**. BLAST results are on the left, while ESTScan results are on the right.

Among 80,686 unigenes, 45,065 (55.9%) were predicted to match CDSs with lengths ranging from 33 to 14,379 bp with an average length of 665 bp (Figure 5). ESTScan indicated that 34,128 of unigenes (42.3%) were predicted to match CDSs ranging in length from 51 to 10,476 bp with an average length of 336 bp (Figure 5).

**Figure 6:**
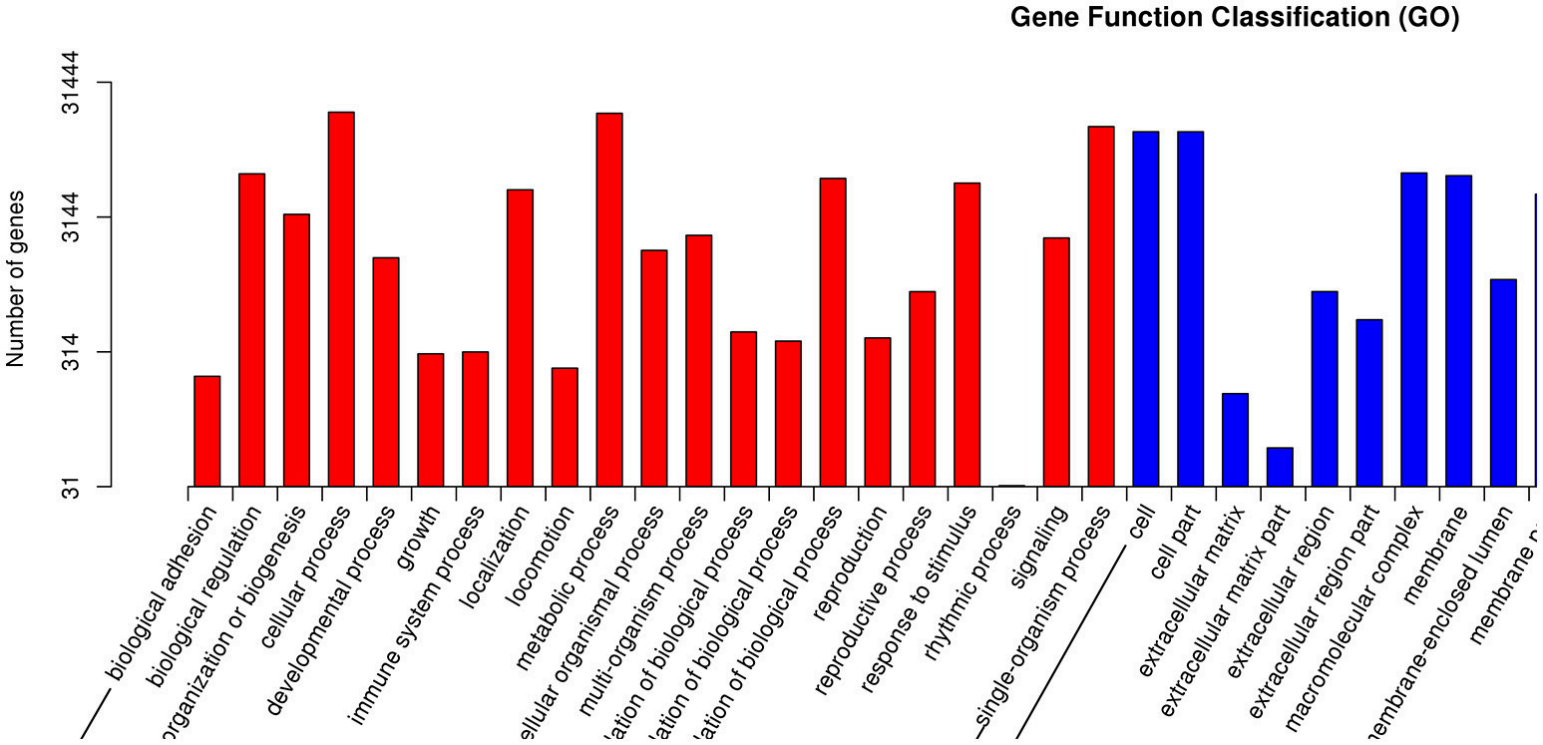
**Gene Ontology classification of assembled unigenes**.

GO is an international standardized gene functional classification system with three ontologies: biological process (BP), molecular function (MF), and cellular component (CC; Wang et al., 2010). Each ontology offers a comprehensive description of the properties of genes. A total of 31,444 unigenes were annotated according to an analysis performed with Blast 2 GO. BP was the most abundantly represented according to GO, followed by CC and MF (Figure 6). Within BP, cellular process (18,850 unigenes, 21.0%) and metabolic process (18,499 unigenes, 20.6%) comprised the majority. Within MF, binding (19,070 unigenes, 46.5%) and catalytic activity (15,383 unigenes, 37.5%) contained the largest numbers of genes, while within CC, cell (13,495 unigenes, 21.4%) was almost as common as cell part (13,492, 21.4%), in agreement with Li et al. (2016).

**Figure 7:**
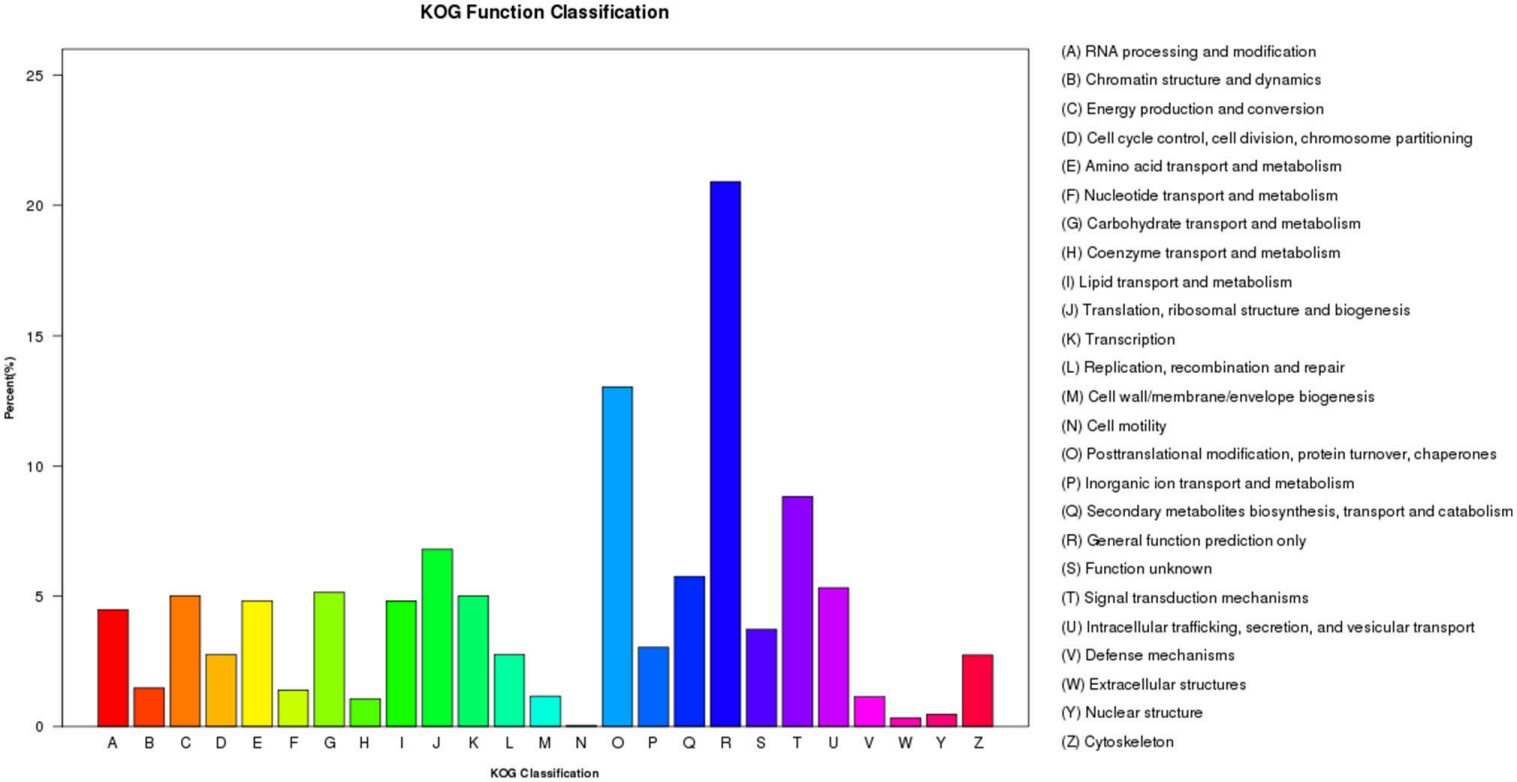
**Histogram presentation of clusters of orthologous groups**.

Within the KOG analysis, 11,778 unigenes were identified to 25 categories. The largest number of unigenes were classified as “General function prediction only” (2,463 unigenes, 20.9%), followed by “Posttranslational modification, protein turnover, chaperones” (1,535 unigenes, 13.0%), and “Signal transduction mechanisms” (1,039 unigenes, 8.8%; Figure 7). However, in *Sorghum*, the category enriched for the most unigenes was “General function prediction only,” followed by “Replication, recombination, and repair” and “Posttranslational modification, protein turnover, chaperones.” Similarly, in sugarcane, unigenes were enriched in the “Replication, recombination and repair” category followed by “General function prediction only” and “Posttranslational modification, protein turnover, chaperones” (Cardoso-Silva et al., 2014). Likewise, Sudan grass grown under exposure to natural daylight, expressed genes that were enriched for “Replication, recombination and repair,” followed by “General function prediction only” and “Transcription” (Li et al., 2016). Thus, different species and different conditions yielded different classifications.

**Figure 8:**
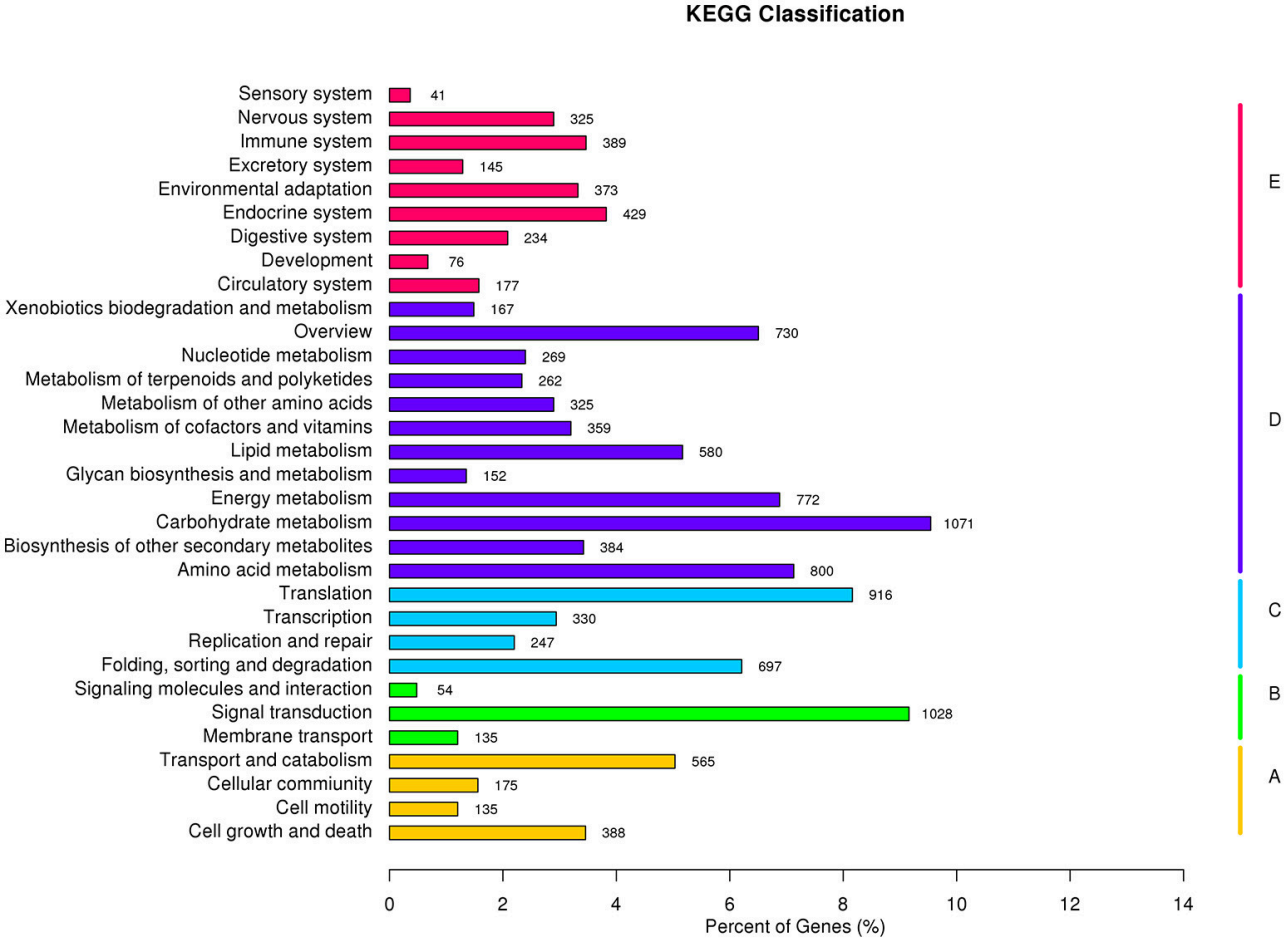
**KEGG classification results: A, Cellular Processes; B, Environmental Information Processing; C, Genetic Information Processing; D, Metabolism; and E, Organismal Systems**.

To better understand the biological functions of and interactions among genes, 11,223 unigenes were classified into 280 KEGG pathways. The pathways included Metabolism, Genetic Information Processing, Environmental Information Processing, Cellular Processes, Organismal Systems, and Human Diseases. Metabolic pathway contained the largest number of unigenes (5,871, 46.1%), followed by Genetic Information Processing (2,190, 17.2%), and Organismal Systems (2,189, 17.2%). Overall, Carbohydrate metabolism (1,071 unigenes, 8.4%) was the most among all pathways, while signal transduction (1,028 unigenes, 8.4%) contributed to Environmental Information Processing (Figure 8). Carbohydrate metabolism was heavily enriched in the KEGG analysis, in accordance with the results of Huang D. L. et al. (2016), as well as a similar study in orchardgrass (Huang et al., 2015).

### DEGs under drought stress

There were 4,903 and 3,466 DEGs identified by comparing expression on the 3rd day to the control expression levels of days 0 and 3, respectively (i.e., L_0_1 vs. L_3_2 and L_3_1 vs. L_3_2 comparisons). Comparisons between Sudan grass exposed to the PEG treatment for 6 days with the day 0 and 6 controls, revealed 9,512 and 6,194 DEGs, respectively (i.e., L_0_1 vs. L_6_2 and L_6_1 vs. L_6_2 comparisons), which indicated that expressions changes that had occurred by the 3rd day were smaller than those occurring by the 6th day. In contrast, the comparison between expression levels on the 3rd and 6th day of the PEG treatment (i.e., L_3_2 vs. L_6_2), revealed only 130 DEGs, with 5 and 125 genes down- and up-regulated, respectively. Few of the same DEGs changed in the 3rd and 6th days, but there were changes among all control samples over time (i.e., L_0_1 vs. L_3_1, L_0_1 vs. L_6_1, and L_3_1 vs. L_6_1; Table 3).

**Table 3:** **Differentially expressed genes across two categories of Sudan grass**.

**Samples**	**Down-regulated**	**Up-regulated**	**Total**
L_0_1 vs. L_3_2	3,319	1,584	4,903
L_3_1 vs. L_3_2	2,355	1,111	3,466
L_0_1 vs. L_6_2	5,482	4,030	9,512
L_6_1 vs. L_6_2	3,746	2,448	6,194
L_3_2 vs. L_6_2	5	125	130
L_0_1 vs. L_3_1	1,962	1,009	2,971
L_0_1 vs. L_6_1	482	212	694
L_3_1 vs. L_6_1	250	814	1,064

**Figure 9:**
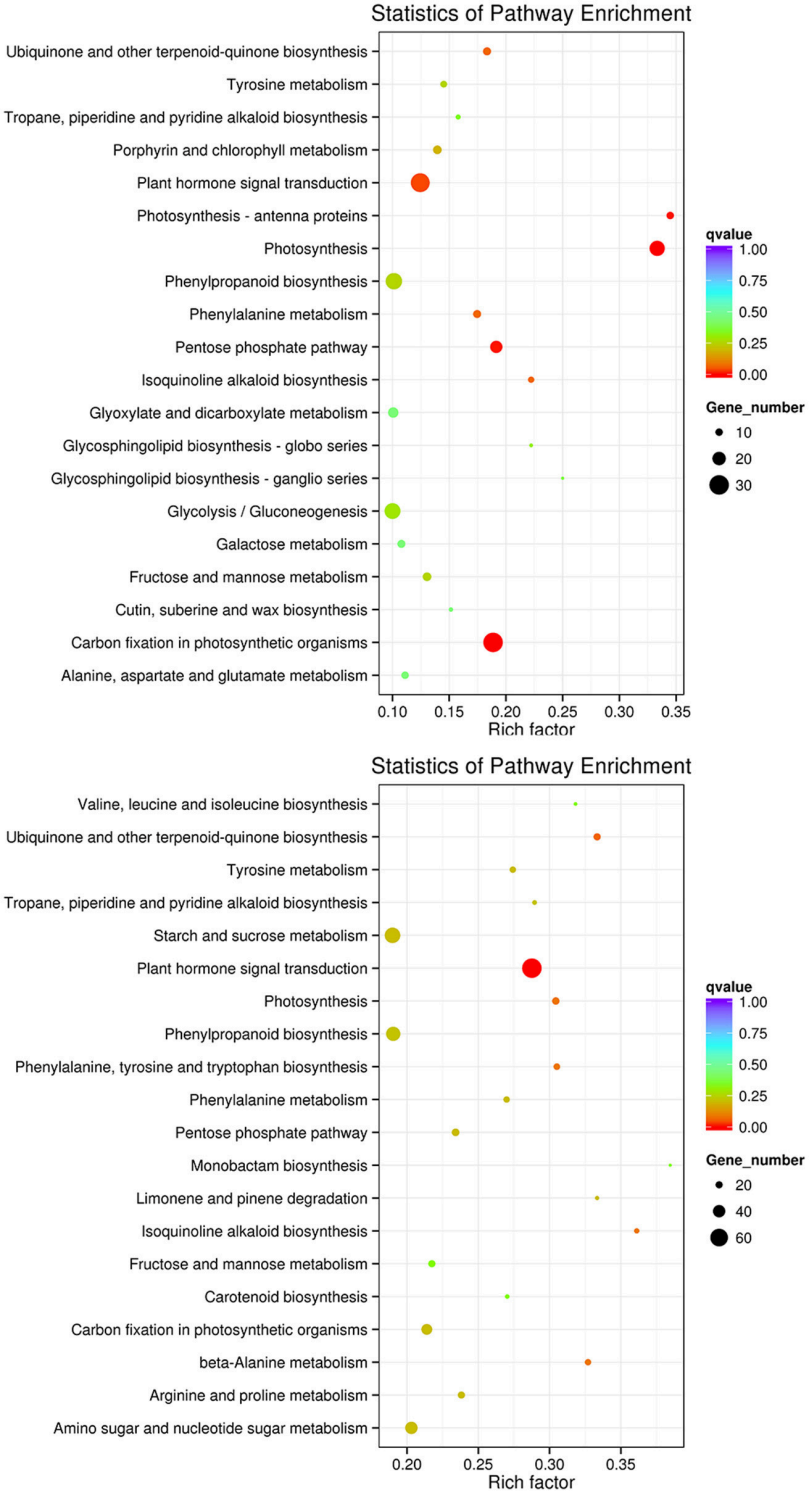
**KEGG pathways enriched for DEGs by the 3rd and 6th days**. KEGG pathways enriched for DEGs by the 3rd day are on the top, while those enriched by the 6th day are in the bottom.

To improve the accuracy of the results, we excluded DEGs that changed in the controls, finding 2,329 DEGs between the controls (0 and 3rd day) and the treatment of 3rd (i.e., L_0_1 vs. L_3_2 vs. L_3_1 vs. L_3_2), which including 1,531 and 785 DEGs down- and up-regulated, respectively. For the 6th days of the PEG treatment compared to the controls (0 and 6th day) (i.e., L_0_1 vs. L_6_2 vs. L_6_1 vs. L_6_2), there were 3,031 and 2,084 DEGs down- and up-regulated, respectively, in total of 5,101 DEGs been founded (Table 4). The DEGs enriched within KEGG pathways indicated Carbon fixation in photosynthetic organisms and Plant hormone signal transduction pathways play important roles in handling short-term drought stress. The next most enriched pathways were Phenylpropanoid biosynthesis, Glycolysis/Gluconeogenesis, and Photosynthesis (Figure 9). Lenka et al. (2011) also demonstrated that the Carbon fixation pathways play an important role in the drought responses of rice. However, under long term drought stress, the Plant hormone signal transduction pathways was most important, followed by Starch and sucrose metabolism and Phenylpropanoid biosynthesis (Figure 9). Huang et al. (2014) declared that the up-regulated genes stimulated by drought stress were enriched in the signal transduction pathway, agreeing with our results.

**Table 4:** **Differentially expressed genes across four categories of Sudan grass**.

**Samples**	**Down-regulated**	**Up-regulated**	**Total**
L_0_1 vs. L_3_2 vs. L_3_1 vs. L_3_2	1,531	785	2,329
L_0_1 vs. L_6_2 vs. L_6_1 vs. L_6_2	3,031	2,084	5,101

**Figure 10:**
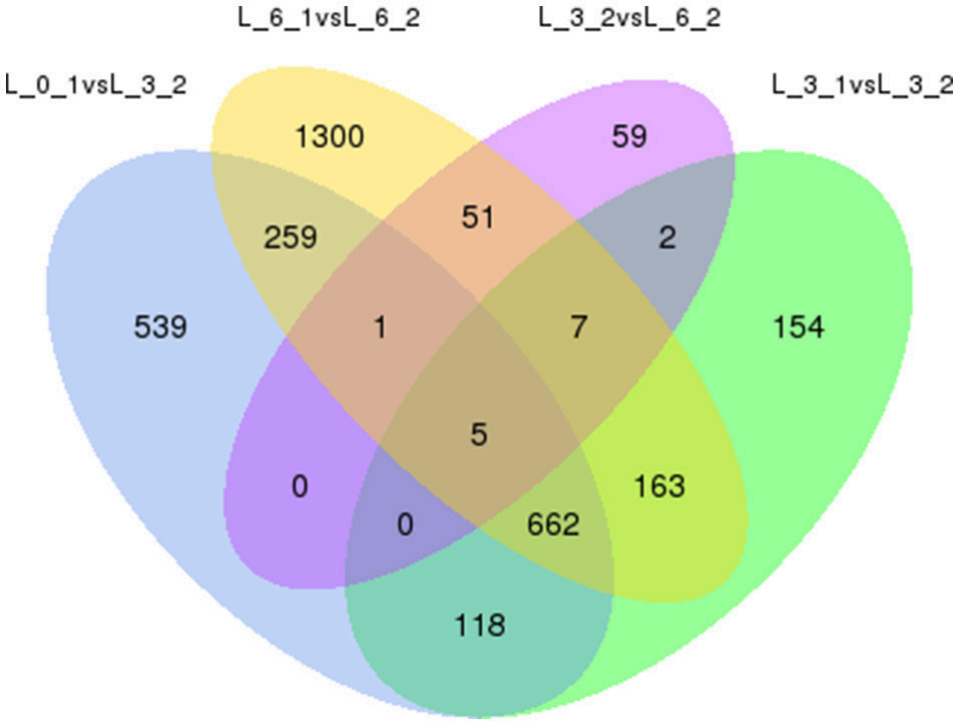
**Analysis of DEGs with drought stress in Sudan grass**. The left one is the up-regulation of DEGs, the right one is the down-regulation of DEGs.

To eliminate expression changes among the all controls (0, 3rd, and 6th day), Venn diagrams were examined (Figure 10), which revealed no genes were down-regulated by PEG stress, while five genes were up-regulated. The corresponding genes ID were c18079_g1, c21893_g1, c33865_g2, c34306_g1, and c35500_g3.

To better understand the functions of these genes, we BLASTed these sequences against NR, NT, PFAM, KOG, Swiss-Prot, and GO databases. The c18079_g1 sequence is 612 bp (Appendix S1), and its NR and NT descriptions indicate it is a hypothetical protein that has been annotated in *Sorghum bicolor*. According to Swiss-Prot, it is similar to *Arabidopsis thaliana*'s defensin-like protein. According to PFAM, it appears to belong to the Gamma-thionin family or Scorpion toxin-like domain. Lastly, GO analysis indicated it has a role in transport or defense response in BP, plasma membrane or ion channel inhibition or activity in MF, and plant-type in CC (Table 5). The first response to the drought stress is reducing water deficit by closure of stomata, which is also called hydropassive closure. When the stomata are open, water evaporates from guard cells. However, stomata are opened by the reversal of ion fluxes (Jouyban, 2012); c18079_g1 may be responsible for mediating this response.

**Table 5:** **Functions of DEGs according to various databases**.

**Gene_ID**	**c18079_g1**	**c21893_g1**	**c33865_g2**	**c34306_g1**	**c35500_g3**
Gene Length	612 bp	1583 bp	3697 bp	2181 bp	1110 bp
NR Description	Hypothetical protein	Hypothetical protein	Hypothetical protein	Phosphosulfolactate synthase-related protein	Hypothetical protein
NT Description	Hypothetical protein, mRNA	*Sorghum bicolor* hypothetical protein, mRNA	*Sorghum bicolor* hypothetical protein, mRNA	Zea mays phosphosulfolactate synthase-related protein	*Sorghum bicolor* hypothetical protein, mRNA
Swiss-Prot Description	Defensin-like protein	–	Uncharacterized AAA domain-containing protein	–	L-ascorbate oxidase
PFAM description	Gamma-thionin Family/Scorpion toxin-like domain	–	ABC transporter/Sigma-54 interaction domain/Magnesium chelatase, subunit ChlI/AAA domain (dynein-related subfamily)/Uncharacterized P-loop hydrolase UPF0079/Parvovirus non-structural protein NS1/ATPase family associated with various cellular activities (AAA)/K-Cl Co-transporter type 1 (KCC1)/Holliday junction DNA helicase ruvB N-terminus/TIP49 C-terminus/Guanylate kinase/AAA domain (Cdc48 subfamily)/Zeta toxin/ATPase family associated with various cellular activities (AAA)/PhoH-like protein/RNA helicase/IstB-like ATP binding protein	(2R)-phospho-3-sulfolactate synthase (ComA)/Small cytokines (intecrine/chemokine), interleukin-8 like	Multicopper oxidase/Multicopper oxidase/Multicopper oxidase
BP Description	Transport/defense response	Protein folding	DNA repair/DNA recombination/regulation of transcription, DNA-templated/threonylcarbamoyladenosine biosynthetic process/transport/photosynthesis/chlorophyll metabolic process/viral genome replication/ion transport/chlorophyll biosynthetic process	Signal transduction/chemotaxis/heat acclimation/coenzyme M biosynthetic process/immune response	Oxidation-reduction process
MF Description	Ion channel inhibitor activity	Unfolded protein binding/heat shock protein binding	ATP binding/transcription factor binding/nucleoside-triphosphatase activity/ATPase activity/RNA binding/magnesium chelatase activity/protein binding/four-way junction helicase activity/transporter activity/RNA helicase activity/DNA helicase activity/kinase activity	Catalytic activity/chemokine activity	Oxidoreductase activity/copper ion binding
CC Description	Plasma membrane/extracellular region/plant-type cell wall	–	Replication fork/membrane/magnesium chelatase complex/transcription factor complex/Holliday junction helicase complex	Extracellular region	Extracellular region/plant-type cell wall
KOG Description	–	–	AAA+-type ATPase	–	Multicopper oxidases

The second gene, c21893_g1, is 1,583 bp (Appendix S1), and it also strongly resembles the *Sorghum bicolor* hypothetical proteins in the NR and NT databases. However, we were unable to find any description of it in the PFAM, KOG, and Swiss-Prot databases, but in the GO database, the functions of the gene were protein folding and unfolded protein binding (BP) or heat shock protein binding (MF; Table 5). Buchanan et al. (2005) indicated that heat shock protein 17.2, heat shock protein 16.9, and HSP 70 were up-regulated by increased PEG stress in sorghum. Several studies reported that drought stress is often combined with heat shock (Wang and Huang, 2004; Mienie and De Ronde, 2008; Grigorova et al., 2011), perhaps explaining the up-regulation of this gene by drought stress in Sudan grass.

The third gene, c33865_g2, is the longest of all five (3,697 bp, Appendix S1). It is similar to the previous genes, which are similar to hypothetical proteins in *Sorghum bicolor*, but in contrast, it matches genes in all of the examined databases. It matches an uncharacterized AAA domain-containing protein from *Schizosaccharomyces pombe* in the Swiss-Prot database, and there were many BP, MF, and CC roles in the GO database (Table 6). The KOG database indicated the gene is an AAA^+^-type ATPase (Table 5). As an ABC transporter-like protein in sorghum, the gene has been shown to be up-regulated under drought stress (Buchanan et al., 2005). Deeba et al. (2012) also showed that activation of the ATP generation in groundnuts can improve drought tolerance. However, the actually function of this gene is unclear.

**Table 6:** **SSR motifs**.

**Repeats motif**	**Number**	**Percentage (%)**
**MONO-NUCLEOTIDE**		
A/T	6,166	
C/G	605	
Total	6,771	38.59
**DI-NUCLEOTIDE**		
AC/GT	806	
AG/CT	1,571	
AT/AT	696	
CG/CG	293	
Total	3,366	19.18
**TRI-NUCLEOTIDE**		
AAC/GTT	140	
AAG/CTT	356	
AAT/ATT	124	
ACC/GGT	506	
ACG/CGT	741	
ACT/AGT	142	
AGC/CTG	1,015	
AGG/CCT	942	
ATC/ATG	204	
CCG/CGG	2,690	
Total	6,860	39.09
**TETRA-NUCLEOTIDE**		
Total	474	2.7
**PENTA-NUCLEOTIDE**		
Total	49	0.28
**HEXA-NUCLEOTIDE**		
Total	28	0.16

The fourth gene, c34306_g, is 2,181 bp in length (Appendix S1). In the NR and NT databases, it matches the phosphosulfolactate synthase-related protein in *Zea mays*. The PFAM database indicated the gene is a (2R)-phospho-3-sulfolactate synthase (ComA) or Small cytokine (intecrine/chemokine), interleukin-8 like gene, whereas GO indicates it has a role in signal transduction, chemotaxis, heat acclimation, coenzyme M biosynthetic process, or immune response (BP); chemokine activity and catalytic activity (MF), and extracellular region (CC; Table 5). Graham et al. (2002) introduced the details of the biosynthetic pathway for coenzyme M. Phosphosulfolactate synthase and ComA catalyze the first step of the coenzyme M biosynthetic process (Liu et al., 2006), and c34306_g may be involved in this process.

The last gene, c35500_g3, is 1,110 bp (Appendix S1), and it is a hypothetical protein in the NR and NT databases. It matched a cucumber l-ascorbate oxidase in Swiss-Prot and a multicopper oxidase or copper ion binding protein in PFAM. In GO, c35500_g3 is characterized as having a role in the oxidation-reduction process (BP), oxidoreductase activity (MF), and extracellular region or plant-type cell wall (CC). Drought stress induces the accumulation of reactive oxygen species (ROS), and therefore ROS-scavenging is stimulated by drought. Osakabe et al. (2014) indicated that ascorbate oxidase plays an important role in scavenging cytosolic H_2_O_2_ (Table 5). The function of ascorbate oxidase was to protect integrated chloroplast proteins, and massive reports have shown that the overexpression of APX2 improved drought tolerance. Increased levels of thylakoid-bound ascorbate peroxidase have been identified in sorghum under drought stress (Buchanan et al., 2005). Dawson et al. (1975) reported that every molecule of ascorbate oxidase contains 8–10 atoms of copper, and this has been subsequently studied as an enzyme model for l-ascorbate oxidase (Ueda and Hanaki, 1984). Thus, this gene maybe involved in the pathway for ascorbate oxidase.

**Figure 11:**
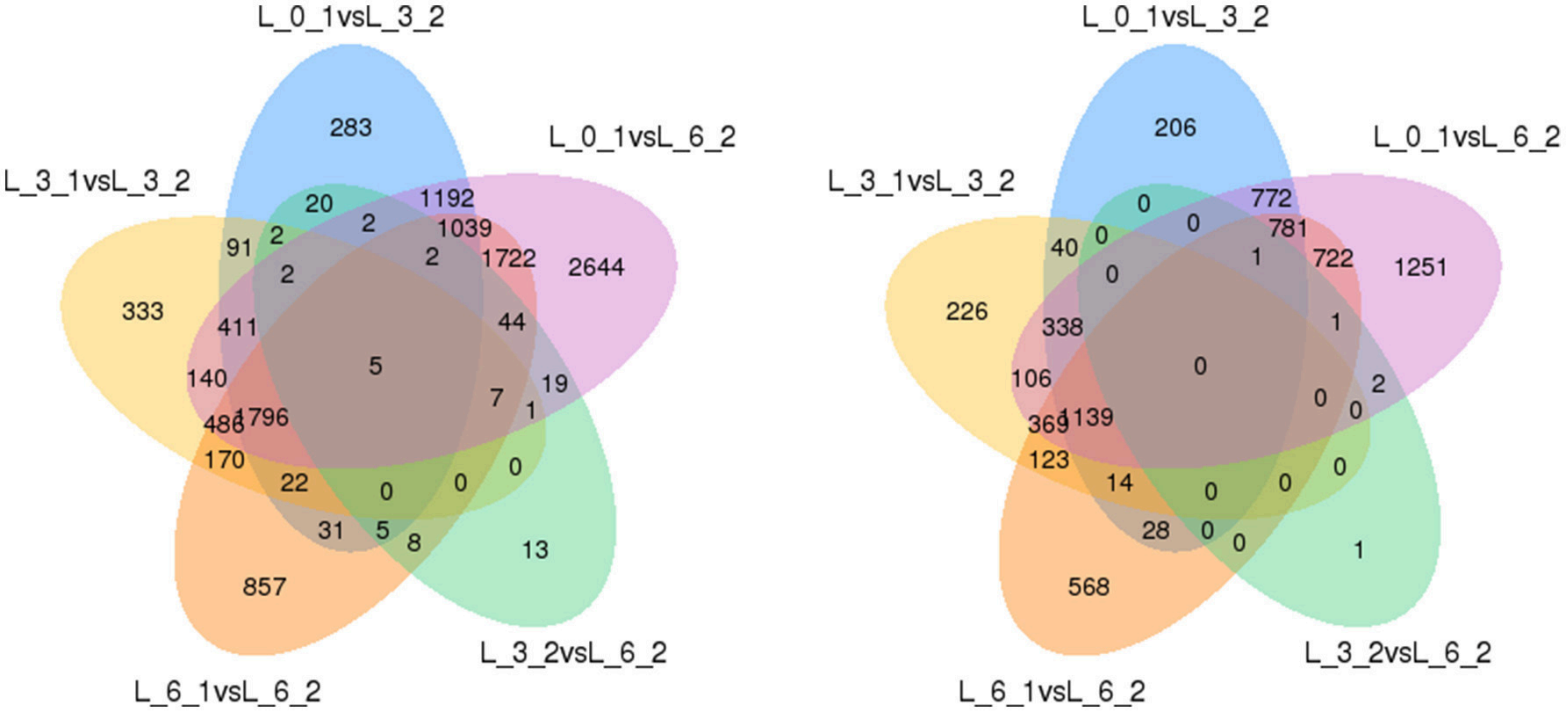
**Venn diagrams of DEGs among 5 combinations**. Up-regulated DEGs are on the left, while down-regulated DEGs are on the right.

Finally, to ensure the accuracy of the Venn diagrams, we used more comparisons to analyze DEGs (5 combinations). These alternative results coincide with the main results. We again found the same five genes up-regulated and no genes down-regulated (Figure 11).

### Development and validation of SSR markers

We identified 17,548 SSRs obtained from 80,686 sequences totaling 75,697,479 bp. A total of 13,574 sequences contained SSRs. Of these, 2,965 sequences contained more than a single SSR, and 866 exhibited compound SSR formation. Trinucleotide repeat motifs were the most abundant among the six types of motifs, totaling 6,860 (39.09%). The second most abundant were mononucleotide motifs, totaling 6,771 (38.59%), followed by 3,366 dinucleotide motifs (19.18%), 474 tetranucleotide motifs (2.7%), 49 pentanucleotide motifs (0.28%), and 28 hexanucleotide motifs (0.16%). The most abundant motifs included mononucleotide A (91%), dinucleotide AG (47%), and trinucleotide CCG repeats (39%; Table 6).

**Figure 12:**
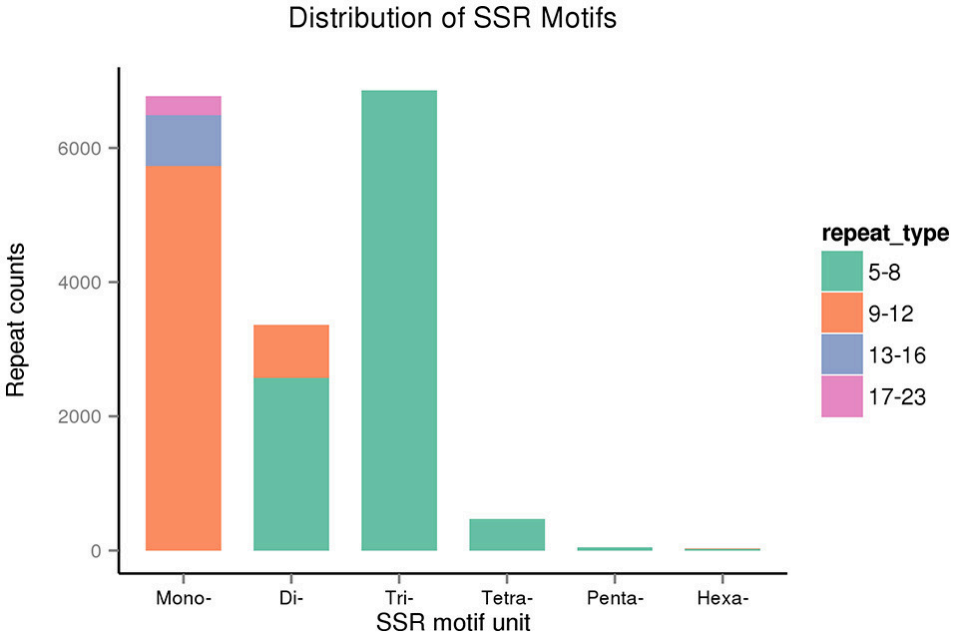
**The distribution of SSR motifs in Sudan grass**.

The repeat counts ranged from 5 to 23. SSRs with 5–8 repeats were being most abundant, followed by those with 9–12 repeats (Figure 12). In sugarcane, trinucleotide motifs are perhaps most common among SSRs, which is consist with our results. Cardoso-Silva et al. (2014) reported that trinucleotide SSR motifs strongly impact the rate of frameshift mutations.

We randomly selected 30 primer pairs for amplification. Of these, 23 primer pairs (76.7%) successfully amplified products from genomic DNA from Sudan grass and *Sorghum* (though the remaining 7 primer pairs failed to generate PCR products). The failure of the primer pairs to amplify may have been caused by the location of primers across splice sites, introns, mutations, or indels.

In total, 124 amplified bands were detected from the PCR products of 23 primer pairs. The products of the primer pairs ranged from 134 to 280 bp. Among the 23 primers, the unigene c24737_g1 amplified by primer pair 23 showed the highest average polymorphism information content (PIC = 0.8304), followed by c25684_g1, which was amplified by primer pair 1 (PIC = 0.7608). However, according to Shannon's information index (*I*), c25493_g1 (amplified by primer pair 9) was the most diverse, (*I* = 0.6851), followed by c24737_g1 (amplified by primer pair 23). According to Nei's gene diversity (*H*), c10226_g1 (amplified by primer pair 7) exhibited the most variation (*H* = 0.5281), followed by c33403_g4 (amplified by primer pair 14; *H* = 0.5169). Among these 23 unigenes, the average PIC was 0.5327, *I* was 0.552, *H* was 0.439, and PPB was 78.18%. Therefore, these 23 unigenes showed high levels of diversity (Table 7).

**Table 7:** **Characterization of 23 novel microsatellite loci for Sudan grass**.

**Gene ID**	**Primer no**.	**Forward primer(5′–3′)**	**Reverse primer (5′–3′)**	**Tm**	**Product size(bp)**	**Repeats**	**PIC**	***I***	***H***	**PPB%**
c25684_g1	1	GGTAGGAGACGGAGGTGAGT	GTCCTCCGATCTGCTAAGCC	60	140	(GCG)6	0.7608333	0.4577	0.3537	100.00
c24947_g1	2	GTTTGCTTGTCCGCTAAGGC	TTGTCGTCGTCCGTTGTAGG	60	197	(ACC)6	0.6325	0.6567	0.4902	100.00
c32362_g4	3	GCAACGGCAAGCAGAGTATG	TTTGCATGCCACAGACTTGC	59	255	(GCA)7	0.4908333	0.5555	0.4925	66.67
c18739_g1	4	ACAGCTAGTTTGGGTGTCCG	CATGGCCGAGGTCAACTCTT	59	140	(GCT)6	0.6357143	0.5622	0.4607	100.00
c30194_g1	5	TCACGCACTGCAGGGTTAAT	ACACGCGTATCAGTGTTCGT	59	134	(A)10	0.7336364	0.6645	0.1948	100.00
c23844_g2	6	CCGCCAAAGAAGACAAAGGC	GTCCAGCTCCATCCTGAACC	60	218	(CGA)6	0.7140625	0.4848	0.4030	100.00
c10226_g1	7	GTGACACGGATCTCACGGAG	GGTGTGGTGAAGAGGTCTGG	60	192	(TTG)5	0.3033333	0.6109	0.5281	33.33
c29788_g1	8	GCGGGTGTTGATGATGCTTG	GCCAAAGCAAGAACGACAGG	60	104	(GCC)5	0.3442857	0.5156	0.5132	57.14
c25493_g1	9	TGCTGAAACTTTGGAATGGCA	CCCCTTGCATGTTCAAGTGC	58	154	(T)11	0.5883333	0.6851	0.4850	83.33
c35250_g7	10	CAATTTTGCCCTCCTTGCCC	CTTCCTCATCTCCTCCGCAC	60	242	(T)11	0.39075	0.5207	0.5045	50.00
c28737_g2	11	AAGATCAAGAAGCCCCTGCC	ATCTGGTGGTACCTCGGGAA	60	143	(GCC)5	0.7382143	0.5887	0.3875	85.71
c22092_g1	12	TGTGACTTTGAGCTCCGTCA	TGCTACCTGAACTCCCCCTT	59	229	(T)11	0.3525	0.5943	0.4448	66.67
c39996_g1	13	TCCGAAATCCTTGTCCCTCT	TCGCCATCGATCAACTCTCG	58	121	(CAC)5	0.453	0.6227	0.5120	80.00
c33403_g4	14	GAGCAGAGGAACCATGGCTT	TCGTCACGCAACAACTGTTC	60	200	(A)10	0.41875	0.5554	0.5169	50.00
c31134_g1	15	AGGTCAGACGCCAAACTGTC	ATCAGCACGTACTTGGTCGG	60	234	(TTC)5	0.1642857	0.4127	0.4668	28.57
c32157_g1	16	CCACATCCGTTCACCCTTCA	GGGTTTGGTTGCTCCGTTTC	59	206	(CT)6	0.57	0.5004	0.4693	100.00
c32633_g2	17	CTCTCTCCCACTCTCTGCCT	AGGGAAGTGCATGCAAGCTA	60	163	(TCG)5	0.715	0.3902	0.3791	100.00
c30552_g1	18	TCCTATGCGCACCACCAATT	GTGACATGATCCCTGGCCC	60	248	(GC)6	0.6275	0.5088	0.4187	66.67
c40055_g1	19	GAGCATCGAGGAGCGTCTC	CTTCTGCGCTCGAAATGGAG	60	183	(C)11	0.23625	0.3183	0.4424	100.00
c23435_g1	20	GGGGCAAAACTGCTGTTGTT	CCCACTTCCTGCTCAACCAG	59	157	(CTG)5	0.66375	0.6276	0.3963	75.00
c32482_g1	21	CGTAATTTGGGCCGCCAATC	CGAACGAATCGCTGAGGTTT	60	275	(A)10	0.626875	0.6272	0.4561	75.00
c17048_g1	22	CATAGTGCCGTAGCCCGTAG	CGGACGACTCTTGGACCAAA	60	280	(GGC)5	0.2615	0.5579	0.4745	80.00
c24737_g1	23	GCAAGATGATATATGGAGTACGGC	TCCCCTTCCTAGAGCTGCTT	59	140	(A)10	0.8304167	0.6704	0.3114	100.00

*Tm, optimal annealing temperature (°C); PIC, average polymorphism information content; I, Shannon's information index of diversity; H, Nei's gene diversity; and PPB, percentage of polymorphic bands*.

SSR makers are extremely useful molecular makers that can be used for genetic linkage mapping, comparative mapping, and many other genotyping applications (Tang et al., 2009). These makers have many advantages including reliable reproducibility, co-dominance, and a high degree of operational transferability to other related species (Wang et al., 2002; Kaur et al., 2011). SSRs have been widely used in sorghum (Sanchez et al., 2002; Mace and Jordan, 2011; Upadhyaya et al., 2012; Hussein et al., 2014), but Li et al. (2016) reported important differences between sorghum and Sudan grass in indel markers. Few studies have tried to use ESTs from sorghum to design SSR markers in Sudan grass. Further genetic analyses of Sudan grass require the development of more SSR makers for the species. Our study has identified 17,548 SSRs that may be used to enhance the molecular breeding of Sudan grass.

## Conclusion

In this study, we used NGS data to analyze the Sudan grass transcriptome under drought stress. We found 2,329 DEGs and 5,101 DEGs under a short- and long-term of 25% PEG treatment, respectively. And we also found five genes related to the drought stress strictly. Notably, those genes were up-regulated in Sudan grass under drought stress. This discovery may facilitate the development of transgenic breeding for drought resistance. We also developed a substantial number of new genetic markers, including SSRs, which can be broadly applied to understanding the genetics of Sudan grass.

## Author contributions

XZ, YZ, and XiaW conceived the project and designed the experiments; YZ, XiaW, and LH performed the experiments; YZ, XiaW, CL, WX, JP, ZL, and HY analyzed the data; YZ, XiaW, FL, XieW, LY, and DP finalized the manuscript; all authors discussed the results and reviewed the manuscript.

### Conflict of interest statement

The authors declare that the research was conducted in the absence of any commercial or financial relationships that could be construed as a potential conflict of interest.

## Supplementary material

Supplementary Image 1 **Sudan grass exposed to 0% PEG (control; top) and 25% PEG (experimental treatment; bottom) at 6th day, with three replicates each**.

Appendix S1 **Five unigenes are differentially expressed among all controls and treatments of “Wulate No.1”**. The c18079_g1, c21893_g1, c33865_g2, c34306_g1, and c35500_g3 indicate the name of unigenes; the bases below the name are the sequences of these five unigenes.
