# Corrigendum: Transcriptomic Identification of Drought-Related Genes and SSR Markers in Sudan Grass Based on RNA-Seq

**DOI:** 10.3389/fpls.2017.01518

**Published:** 2017-08-29

**Authors:** Yongqun Zhu, Xia Wang, Linkai Huang, Chaowen Lin, Xinquan Zhang, Wenzhi Xu, Jianhua Peng, Zhou Li, Haidong Yan, Fuxiang Luo, Xie Wang, Li Yao, Dandan Peng

**Affiliations:** ^1^Department of Grassland Science, Animal Science and Technology College, Sichuan Agricultural University Chengdu, China; ^2^Soil and Fertilizer Research Institute, Sichuan Academy of Agricultural Sciences Chengdu, China; ^3^Sichuan Academy of Agricultural Sciences Chengdu, China

**Keywords:** Sudan grass, next-generation sequencing, differentially expressed genes, simple sequence repeat markers, PEG

In the original article, there was a mistake in the legend for Figure 10 as published. The part of this graph which on the right is missing. The correct legend appears below. The authors apologize for this error and state that this does not change the scientific conclusions of the article in any way.

**Figure 10:**
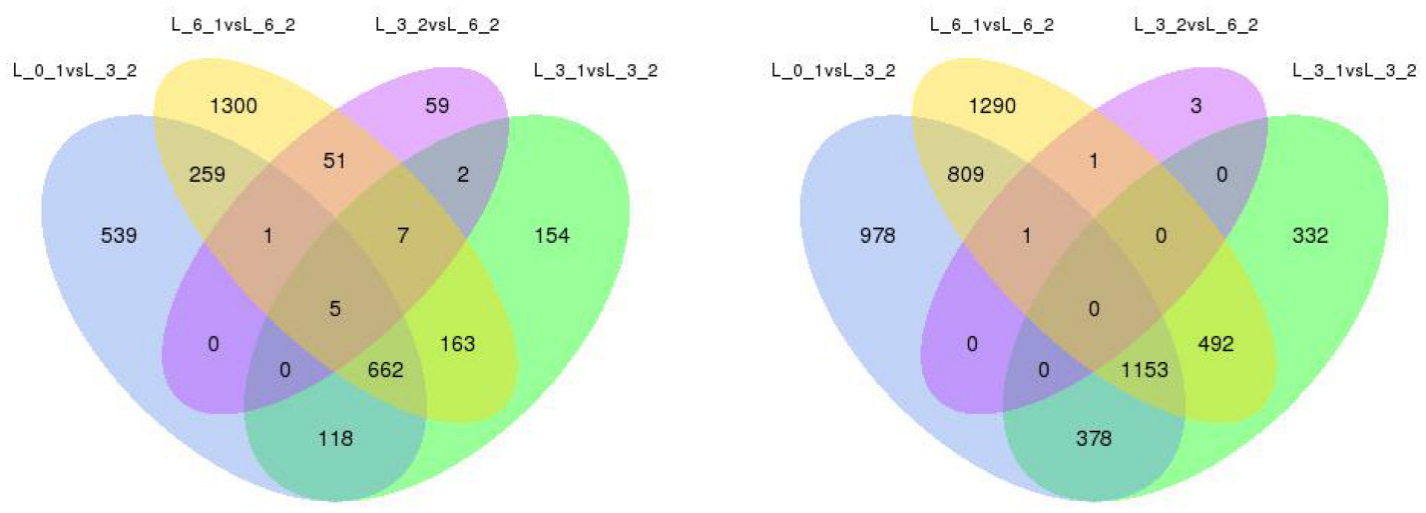
Analysis of, DEGs with drought stress in Sudan grass. The left one is the up-regulation of DEGs, the right one is the down-regulation of DEGs.

## Conflict of interest statement

The authors declare that the research was conducted in the absence of any commercial or financial relationships that could be construed as a potential conflict of interest.

