# Supplementary figures and images for: Transcriptomic Identification of Drought-Related Genes and SSR Markers in Sudan Grass Based on RNA-Seq
Source: Front Plant Sci. 2017 May 4;8:687. doi: 10.3389/fpls.2017.00687 (PMC5415614; doi:10.3389/fpls.2017.00687)

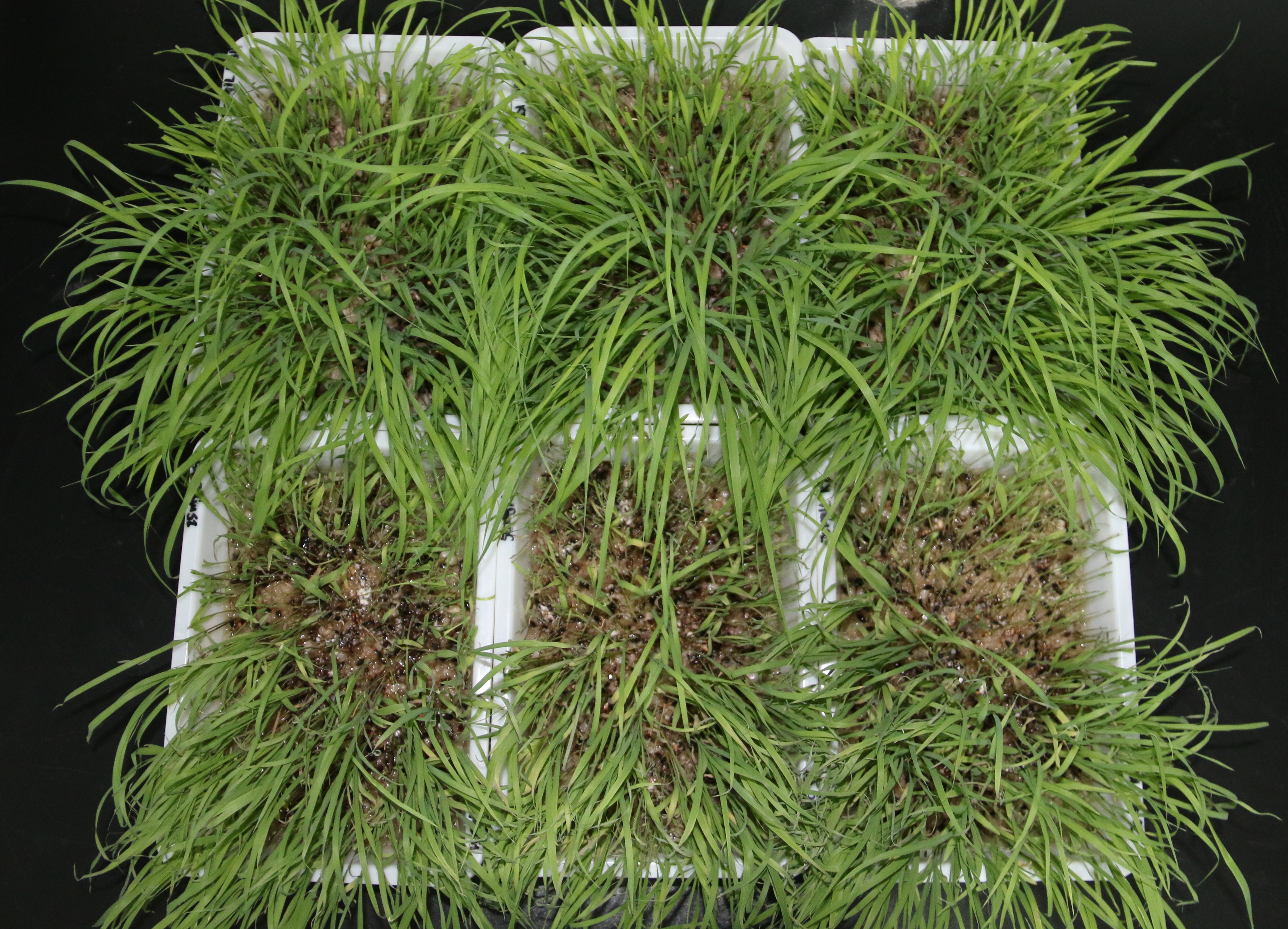

Supplement: Supplementary Image 1 — Sudan grass exposed to 0% PEG (control; top) and 25% PEG (experimental treatment; bottom) at 6th day, with three replicates each. [file Image1.JPEG]
