# Supplementary material for: Transcriptomic Identification of Drought-Related Genes and SSR Markers in Sudan Grass Based on RNA-Seq
Source: Front Plant Sci. 2017 May 4;8:687. doi: 10.3389/fpls.2017.00687 (PMC5415614; doi:10.3389/fpls.2017.00687)
Supplement: Appendix S1 — Five unigenes are differentially expressed among all controls and treatments of “Wulate No.1”. The c18079_g1, c21893_g1, c33865_g2, c34306_g1, and c35500_g3 indicate the name of unigenes; the bases below the name are the sequences of these five unigenes. [file DataSheet1.DOCX]

Appendix S1 Five unigenes are differentially expressed among all controls and treatments of “Wulate No.1”. The c18079_g1, c21893_g1, c33865_g2, c34306_g1, and c35500_g3 indicate the name of unigenes; the bases below the name are the sequences of these five unigenes.

c18079_g1

TTTCTCTTATATTAGGAGTAGATCTCAACGCATTGATATGGAGAAACCGAAACATTGAGGGGAAGAACAGAGGAAACAGCA

GCTAGCAGCACGCGGAGGCGGCACACACTACTATAGACCGATCGATCCACGACTCAACAAACGACAAATATAACTTGTGAC

ACCAGCTGACAAAAAGGTCCAAAAACAAACCACACAAACACACTTCTTCTTATTATTCATGGACGGTTCTCGCGATCAACG

CGCGACGACACGGCAACAGAGATCGAAAACGACGGGCCCGCCGCATGCACTCGCCGCCGGGCAGGCATGCATGCATGCTC

CCCCGGCCGATCGATCGAATCAGTTGCGGCAGTGCGTGAGGCAGAAGCAGCGGCGGCGGAAGCCGCGGCACTTGCCGTCG

GGGAAGCCCTCGGTCCTGCAGACGTTGGCGCAGTTTTCCCGGCGCACGCAGGGGCCGCGGAACCTGTGGCTCTGCGACTG

GCACGTCCTCGCCTCCGCCACCGCCACCGGCCCGATCTCTGTGGCCACGAGCAGCAGCATGACGAGGAGGACGGCCGTGA

AGAGCTTGCGAGAGAGCTCCATCGCTCCGGTTCCTTCTTCTCAGCGGCG

c21893_g1

GAATTTCAAGCATGCCAATCATTGATACAGAAGTTCCATTACACAATCTGACAATCAGGCCTTTGAAATGTTGTATCAAG

CACACACGAACTGTAGAGCCTAGAGCGGCAACATCAGCTTTGATGGCATACTCTGATGACAACTCGTTTTTGATGGCATA

CCCTTATGTACAAATTGTCTTGGCACAACTCATCTCTACCATGGCAGGCAGGAGACGAAAACCAAACAAAACGAAACAAA

AATCCCTTCTTATGAGTGGCGATCCTACGCGACTACCCCTTTGTGGCCTTGTCCTGCAATCTTCCCACCTCACCTTCTTT

CACCCCCCACAATGCAAAGCAATTGCAAATCAGACAGAGTCTGCCCATGGCGGACGTTGGCTCTGACGGGTTCCTAGTTC

CTACTCTCCCTCCATTTCCACGAGCTAGAAAACCCATTGCTGCCGGATTATTATGGATAAAAGCTCGACAATTTATTTAT

CACCATCATCGTGAGAAAGCCGTCCGGCTGAAGCACGCCGCGTCACCGAACCCCTGGGGATGCATGCGTGACGAAGCAGT

ACTCTGCTCTTTGCTGCTCGGTTTGCCCGATGCCCCACCGGTGTAGAAGACACTGGGCTCAGGTTGTGGTGGCGCGGTGA

AATCCTTGATCATCCCGTCCAGCATGGACTGCAGATCGGCCAAGCTGTACACTGGCTCCTCTCTCCCCACGGTGGCCATG

AGCGAGACCATCTCCTGAACGAAGTCGTGGAATCCCTCAACGTCCTCCTGGTCGTCGTCGAGCGGGTCGTACATGCCGGC

GTCGTACAGCGCCCTCCTCTTGTCATCCGACAGCACTGCGCGCGATTGACACATGCAAACGGAGGAGAAGAGGATGATTA

GCAAGCGCGATCAAGGTCGTCCAGTCCAATGCATCGGCTCGGCATCCATTAGGTGCAGGGAGTTTGGGTCCTCTACCTTG

GTACGCCTCGTGTATCTGCTGGAACCTGATCTTGGCCTCCTCCGCTCGCGACGCATCCACGCGGCCGCCGGCGATCTTGT

CCGGGTGCCACCTCTGCATCGTCGTCAGAGAAAAGCATGTGTCATGCTCTGTTTCTTGACATCCTCCGGAGCCAAATGCA

CAATACCAAACCAAAGCGATTGTGTTTTTTATCGATCGCAGGAGTTGCTAGCTAGGATCGAGTAGTACTCATACCATGGC

GAGGCGGTGGTACGCGGCGCGGATCTCGGCGGCGGTGGCGCCCGGCTGCACGCCGAGCACCGCGTAGTACGACTGTGCCT

GCGGCTGCATCCTCCTCCCGCTCATAACGGGCACTAATCCCTCTCTAGTCTGCCAACCAATCTCCCGAATCTCTGGGCAC

TTGCACCCCCACCACCACCTCTCGCGCGCTGGTGCAAAGCTAGAGCAACGATCGCCTCTAGTAGTACGGAAGGGACCCGG

GCGGGACGGCGGTGGATGAATTGATGGCGATGGCGACGACGGAAAGCGGCGGAGGGTGGCAGCCTGTATATATATACAGA

GCACGAGCACGACGGTACAGGCACAGGAGCGAAAAATATCTGGGCGGACAGAAAGGAGCAGAG

c33865_g2

CACCCAAAAAAAGAATATGGAGACAGAGGTTCCGGGTGGAAGAATGCATGCCACATTGCCACCACCCCACACCAACCACA

CCACAGACGGCCTTGGAAAGGAAAAGAAAAGCTAGTGGTACCGTACGTGCGTGTATCGCTATATCACACACCACGTACGC

AGGCCGCGTGCCGGCGCGCCATTGCACTGCCTGCCTTGTGTGCCTGAGCAGAGACGCGGAGGGGAGGAGGAGACAGACAG

GGCAGCGAAGGACCGAGAAGCTAGCAACGGCAACGAGCAGAGGCCCGGCACTTGCGCTCCGCGTCCCCCTCCCTCCGCCA

CCTCTCCCGCTCTCCATTCCGCCACCCACACCACCACCCGTTGCTCGCTGCCGGCCGCCCACGCCACGCCTGCCTCTTCT

CTTCCATCCTCCCTCCCGTGCCCCGCCCCGGCCGGACCTCTTAATTTTAGCCGCCGCCGCCACCGCCGTCGTCGTTGGAT

CGTGTCGTGTCGATCGCCGCCGCCGGCCGGAGAGCAGCAGGCAGCCCGCCCGCCATGGAGGGGAAGAGCGTGGTGATGTC

GGCGCTCGGGATCGGGATCGGGGTCGGCGTCGGCCTGGGCCTCGCGTCCGCGCCCTGGGCCGGCGCCTCCGCCTCCTCCA

GGGCCGCGGGGGTCACCGTCGAACGGGTGGAGCAGGACCTCCGCCGCCTCCTCGTCGACGGCAGCAACAGCAAGGTCACC

TTCGACGAGTTCCCCTACTACCTCAGTGAGCAAACACGTGTTGTGCTGACAAGCGCTGCGTACGTTCACTTGAAGCAAGC

AGAGATTTCAAAGTACACCAGGAATCTTGCTCCAGCCAGTCGCGCAATCCTGCTGTCAGGTCCTGCAGAGCTCTACCAGC

AGATGCTTGCCAGAGCGCTTGCCCACTATTTTCAAGCGAAGTTGCTGTTGCTAGATCCTACTGATTTTCTTATAAAGATC

CATAGCAAATATGGCACTGGTGGCAGTACAGAGAAGACGTTTAAAAGATCAATCTCCGAGACGACGCTTGAACGTGTGTC

GGGATTGCTTGGATCTCTTTCAATTATTTCACAAAAGGAACAGCCCAAAGGAACTATACGTAGACAAAGCAGCATGACGG

ATGTCAAATTAAGGAGCTCTGAAAGTATGACCAACTTACCAAAGCTCAGAAGAAATGCATCTACTTCCTCTGATATGAGT

AGCATGGCTTCGCAAGGACCTTCAACTAATACAGCTCCTCTTAGACGCACAAGCAGTTGGAATTTTGATGAGAAAATTTT

AGTGCAAGCGTTGTACAAGGTTCTGCATTCAGTGTCAAAGAAGTACCCAGTTGTACTCTATATAAGAGATGTTGAGAAGT

TTCTTCACAAGTCCCCAAAAATGTATCTCCTGTTTGAAAAACTACTAGACAAGCTTGAAGGGCCAGTGTTAGTCCTCGGA

TCAAGGATTGTAGATATGGACAGTGATGAGGAGTTAGATGACAGATTGACTGTTCTGTTCCCATATAATATAGAAATCAA

GCCACCTGAAAACGAAAACCACCTTGTAAGTTGGAACTCCCAATTAGAAGAAGACATGAAGATGATTCAGTTTCAAGATA

ACCGAAATCATATTATGGAAGTCCTTGCAGAAAATGATCTTGAATGTGATGATTTAGGCTCGATTTGCCTATCTGATACT

ATGTGCCTTAGTAAGTATATAGAAGAGATTGTGGTGTCTGCGGTTTCATATCACTTGATGAATAACAGAGATCCAGAGTA

CCGAAATGGGAAATTAGTTCTATCCACAAAGAGCTTGTCCCACGCATTGGAAATTTTTCAAGGCAACAAGATGGGCGATA

AGGACAGCATGAAATTGGAAGTGACGGATGGTGCTTTAAAGGCTGCTGAAAAGGCAATTGCTCCAGCCACTGCAAAATCA

GAAACAAAACCTGCAACGTTGCTGCCACCAGTTCGACCTCCGGCTGGTGCTGCTGCTGCTGCTCCCTCCAGTGCCCCTGC

TCCTAGAGTTGAGAGCAAAACAGAACCGGAGAAGAAGGATAATCCACCTCCAGTTGCAAAAGCACTGGAAGTGCCACCAG

ATAATGAGTTTGAAAAGCGCATTAGACCAGAAGTGATACCTGCTAATGAAATTGGAGTTTCATTTGATGATATTGGTGCC

TTGGATGATATCAAAGAATCCCTTCATGAGCTTGTCATGCTGCCTCTTAGACGACCTGACCTCTTCAAAGGCGGTCTTCT

TAAGCCCTGCAGAGGTATATTACTTTTTGGTCCTCCAGGAACCGGCAAGACTATGCTTGCCAAGGCCATAGCAAATGAAG

CTCAAGCAAGTTTTATAAATGTCTCTATGTCGACTATCACATCAAAGTGGTTTGGTGAAGACGAAAAGAATGTTCGAGCA

TTGTTCACATTAGCTGCTAAAGTATCACCAACCATCATCTTTGTCGATGAAGTTGATAGCATGCTTGGGCAGCGGAACAG

AGCTGGAGAGCACGAGGCAATGAGAAAGATCAAGAATGAATTTATGACACACTGGGATGGACTCTTGTCAAGACCAGATC

AGAGAATTCTTGTTCTTGCTGCGACTAACCGGCCTTTTGATCTTGATGAGGCTATCATCCGTAGGTTCGAGCGAAGAATC

ATGGTAGGTCTGCCATCCATGGAAAGTCGGGAACTCATAATGCGGCGACTTTTGTCAAAGGAGAAAGTTGATGAAGGGCT

GGACTATAAGGAGCTAGCAACCATGACAGAAGGATATAGTGGAAGTGATCTCAAGAACCTGTGCACGACGGCAGCATATC

GCCCTGTGAGGGAGCTAATCCAGAGGGAAAGAAAGAAGGAGCTGGTAATCAAACCAAACCACCAGCATCTTAAGAATTTA

ATTCATGATGATTTTTTTGAATGAATTCACTATGTAAGCTTCATCTTCAATGTCTTCAGGAGAAGATGAAGCGTGAAAAA

GGAAAAACTCCATCGGATCTTCCAGAGAAGAAAGAAAAGGAGGAGACCATCATTCTAAGGCCGCTGAACATGACAGATCT

GAAGGAAGCGAAGAACCAGGTGGCTGCAAGTTTCGCTGCGGAAGGCGCCATAATGAGCGAGCTGAGGCAATGGAACGAGT

TGTATGGTGAAGGAGGCTCCAGAAAGAAGCAGCAATTGACATACTTCCTCTGAGCAACATGAACATCACACGCAAAAAAT

GTATGGACATAATTTGCAAAAGTAGAGTAGAGCACAAAATAGCCATGAGGAAAATAGGCCGAACATGTATAAGAACATAA

GAAACGGGGAGTGTTAGGGGACAAGAGTTCAGGCCGTAAAATCCAGTTTCTGGTGTAGGACAAGGAAGAACAATTTTTAT

CTCCACCTAAATTCCAGTTTGCCTGTGCTGGTTGTTGTATTACACTCTAATAATGAATCTGCAGTGCAGATTGCTCTTTT

TTCGTTGTTTTCTCTTTGCCTGGTCGCATCATCCTTTTGTATAGGTATAAACTGGTAGGTTGTGGCTAGCAGCGAAAGGT

TTACGTAGCAGCTTATATCTATATAATGTAAGCAACTTCTTGTCCATGTGGATGCGTGAGGTCAAACAAAACCTAGAGCA

CATGAGTCCTTTTCTGTTTGTTTTAACACAGGTGCAGTACAGATGCGTGAGGTCTCTTTGCTCTCTCTCATTTTCTGAAC

TTTACTCTCTATATATG

c34306_g1

GTGTCCATGTGACTGCTGCCCGATTAATGTTTGCTTGATACCAAACAACCCTAGATAACTCACTACACGGAGATAGTTAA

AAGCAAAACTACAAACATACGACAGATTCAGACCAACATATAAACTAGAAACATCTCTGTTTAGCACAGATGTCTCCATT

CTTGTTCCTACGCTGCAGCACTATGATGTGCCTAGGTTTCTTGTACTTTCTTGTACTTTCTTGTTCTTTTATCATGGAAA

ATTTGCATTACCACAGTGGAAAGACAGTACATAGAAATATAGTTCTTGTCAGCTCTGTTCTACATAATCCTACCATTATT

GTTTCGATGTCAAGAACAAATTGCTAGCTCCTGATCCACGTTGGTTTGATCCAACGAGCTGCAAGATGACGGCAAACATG

AAGTCCGAGCTGGAAAACTATTTTCACTATTGTACACCTGTGTCCTGCTTTTGAAACCAGTAAATAACCGAAGTTCATGC

AGCGACGTGCTCTCATGCTCGAAAAACAGAAAATGCCGATATTCACACGCATACAACCGCACAACCATGAATCCAAGAAT

CTGGCTCATTGATGCAATGAACACAAACGCCCATACACTACTTACACCACAGAGCTGATTATTACAGTAGCGTTACTCAT

AACATAACAGATGCCTGCTCGATGGTGGTTACATGGAGGCCATAGCAAGCTGTGCGAGGCTCACAAGTCCATGTAGAAAC

TTAGCTTGTCACAGCTCAGGATACGGGAACGATCATTAACCCATTACATCAGGAAGAACGGCGAGGCAACATGGGAGCCC

AAGTTGCTTCTGAGCATGTTGAAGCCCCGGAGGCGCTCCAGATTCATCACATCGGAGTGGTCAACGAAGAGATTTACCCT

TGGGCCATATCGTCTGACAAACCACTCTGAGGTTTTGGGATTGGAAGCTTCAAACATGGTCTTATCAAGTCCGAGCCGGC

CTACAATCTTGGCAATGATGTCTGTCCTCAGAGATTCAGCATGCTGGCAAACATCATCGGCGTCGATCATGATCATATCT

GCGCCTGCTTCCAAGCATCTCTCTGCCCTCCTAATCAGCAGGTCAACATCTTCAACTCTTTCTGCAAGAGTGGACACCAA

ACACACGTGTTTTGAGTAAAAAAAAAAGAATTTGATTACAGGGAGAAGACTTCACCCTGGCTTTGACTTTAAGGAGTGCC

CAATCCTGGCTGGCTAGAAAACTCGCTGAAACCAGTTCTCAGGAGTCAGGAAAGCTGGCTGGTGAGTGGCGAGCCTCTCT

AATGGAGGTTCTACCAATTGAGCTATCATTCAGTTCCCAGCACAGATCGTCCATAAAGGGACAATAAGTTTCTTCACTTG

CTTCCTTAGAAGAGTATGACTCCTATATTAGCTATTGTTGTGCTACTTGTAAACAAATAATGTGTACACAGACAACAAAG

AAATAATGATTGAGATTTTCAGACTTCTGACAGTGACAGCTAAGTTGTGCTTTTGTGGTTTGAGGTTACATCAGATTGCC

TCATCAATCAATAAAATTCGACAGACCCAATACTTTTCATCAAGTAATGTGTACATAGCTAAGGCACTAAAGTTTTAGCC

CAAGGCATGTTTTGCAATACCAACCCACAACTTTGTTAAAAGGAAGTTTTAGAAAATTTAAAAAATAAAGGGTGAATTAA

GCTTTGAAATACATTTATATTTGGTAAAAGTACATCATATACCAGTGCCCATGATGAAGAGGGCACAAAGGACAGGAGCA

AAAAGAACCAAAATAAAATAAATACCTCACTTCAACTTAGAAATGCCAAAGCCATTGGATTATGTTACATTATTGACTTT

CAAGACCTGCTAACCGTAACATAAGACAGAACGGCCAAAAACAAAGCAACAAGATTACTCCATGCATCCCCAAGCTAATC

ATTTCAAAGATATTTTGTTCTCTAATTGGTTTTAAACTCCGTAATGTATTATTAATTATTTATTGGTGAACTCAATTGTA

AACATTATTTCCGCTGCAAACTCTAGTGTATGTGATGTGTATTTATTAATCTATTACAGTCTTGGTTGTGATGTGGATTT

ACCGAGGTCCTTCGTGACACTCGGCGGACTACCGGGTTTATATAAGTGGAAGTGTGTGTGTGTGTGTCAATGTGTGTGAG

AGAGTTTGTGTGTTGAGTGCT

c35500_g3

ATCTTGCGGCACATGCATGGAGCACTAAATATAAATAAAAGAAATAATTAATTACAATTTACCTGTAATTTGCGAGAAGA

ATCTTTTAAGCCTAGTTCGTTTATGATTGGATAATATTTATCAAATACAAACGAAAATGCTACAATATTTATTTTATAAT

TTTTTTTTGAACTAAACGAGGCCTTATATTACCCCTTGGACCTGCACGTTGAGAGCGGAGAGGGAGGTGGTGCTGGCGAT

CCGGAGCCTGTACGTCTTGCCGGGGTCGACGTCGAAGACCACCGGCGAGCACTGGCTGCGCTCGCAGAAGGGACCGCACT

CGCTCCGGCGGATGAGCTCGCAGCGTTCGCTCTTGTCCCCTTCCTTGCACAAGGCGTCCCGTTTCCTCCTGTCGCAGGCC

CTGCGGTCGCCGGTTATTCCCAGGGAGCACCCGAACTGGCCCCTCCCGTTGATGAGAATCGTCTGAGGCTCGCCGACCCA

CTCCCAGTGCTTGTCCTTGCCGTCGAGCCCGGCAGCCTGCGCGTACACGTTCTCGTGGTACCAGTCGCTGAGCAGCATGT

TGAGCTCGCCGTCGTACTCGGCCGCGAACGGCTCCGGCTGCTGCTCCGTGCCGTTCACGATCAGCGAGCCGTAGAGACCC

GCCGCCCGCTGCATCCCGAAGTGCCCGTGGTAGAAGAAGGTTCCAGGCTTGTCAGCTATGAACTCGTAGGTGAACCTCTC

CCCTGAGTTGATTGGGCACTGGGATATCGACGCCGTTCCATCAGCCCACGGTGTGCCGATCTGTCTGATTCCATGCCAAT

GGATGACGACGCCCTCGGTATGTAGGCTGTTGGTCACCTCGACCCTGATGAGGTCGCCGGCGTTGGCACTGATCGTGGGG

CCGGGGAACTCGCCGTTGATGCCGATCATCACGCTCTGATGGCAGTCCGGCGCCCACATGATGTACTCCACGTCCCACGT

GAAGTTCCGAGTCGCCGGAGCCGGAGAGGATGGAGATGGGGCTGCAGCGACAGACTGGGCCGAGACCGGCATCATCAAAG

CGCAACAAATAAGCAGTTGCACTGCTAGAGAGACATAGGGCTTGCCCATCGTAGCTCGAAGACAGGAGCA
